# scTAM-seq enables targeted high-confidence analysis of DNA methylation in single cells

**DOI:** 10.1186/s13059-022-02796-7

**Published:** 2022-10-28

**Authors:** Agostina Bianchi, Michael Scherer, Roser Zaurin, Kimberly Quililan, Lars Velten, Renée Beekman

**Affiliations:** 1 Centre for Genomic Regulation (CRG), Barcelona Institute of Science and Technology (BIST), Barcelona, Spain; 2 Universitat Pompeu Fabra (UPF), Barcelona, Spain; 3 Centre Nacional d’Anàlisi Genòmica (CNAG), Barcelona, Spain; 4 Institut d’Investigacions Biomèdiques August Pi i Sunyer (IDIBAPS), Barcelona, Spain

**Keywords:** DNA methylation, Epigenetics, Single-cell profiling, Multi-omic analysis, Hematopoiesis

## Abstract

Single-cell DNA methylation profiling currently suffers from excessive noise and/or limited cellular throughput. We developed scTAM-seq, a targeted bisulfite-free method for profiling up to 650 CpGs in up to 10,000 cells per experiment, with a dropout rate as low as 7%. We demonstrate that scTAM-seq can resolve DNA methylation dynamics across B-cell differentiation in blood and bone marrow, identifying intermediate differentiation states that were previously masked. scTAM-seq additionally queries surface-protein expression, thus enabling integration of single-cell DNA methylation information with cell atlas data. In summary, scTAM-seq is a high-throughput, high-confidence method for analyzing DNA methylation at single-CpG resolution across thousands of single cells.

**Supplementary Information:**

The online version contains supplementary material available at 10.1186/s13059-022-02796-7.

## Background

DNA methylation (DNAm) at CpG dinucleotides is an epigenetic mark extensively modulated in health and disease. To date, DNAm has primarily been investigated in bulk samples, which hinders the study of rare cell types, differentiation processes, and cellular heterogeneity. Single-cell DNAm (scDNAm) methods can overcome these limitations but currently require prohibitive sequencing efforts to cover the 28 million CpGs in the human genome. Available techniques with a cellular throughput of more than 100 cells produce sparse datasets, where only 1–7% of the investigated CpGs are randomly covered in a single cell [1–6]. Notably, recent progress in scDNAm methods substantially increased the number of CpGs to up to 30% [7], but the methods still require prohibitive sequencing effort to reduce the dropout rate. Most CpGs in the genome are not informative to assess at single-cell level, as they are either constitutively (un)methylated or display no variable methylation within the tissue of interest [8]. For instance, less than 4% of CpGs show a DNAm difference of more than 50% across the differentiation process of human B and T cells [9, 10] (Additional file 1: Fig. S1). Bulk DNAm data is available for most human tissues and cell types [11] and can be used to identify CpGs with variable methylation [12]. Additionally, single-cell, genome-wide reference DNAm maps are emerging and serve as a rich resource for identifying variably methylated CpGs [13]. However, generating these maps are multimillion-dollar efforts that need to be complemented by cheaper methods accessible to individual investigators and for future clinical applications. Targeted approaches that focus sequencing on variable CpGs can efficiently dissect intra-tissue DNAm heterogeneity, and complement single-cell genome-wide methods. In most human tissues and cell types, targeted methods can capture a large fraction of the variation observed in whole genome data (Additional file 1: Fig. S1), but have so far been limited to a throughput of less than 100 cells per experiment, and less than 60 investigated CpGs per cell [14–16]. To fill the existing gap of high-throughput, targeted scDNAm methods, we developed scTAM-seq, a targeted bisulfite-free method for profiling up to 650 CpGs in up to 10,000 cells.

## Results

### scTAM-seq profiles the methylation states of up to 650 CpGs using Mission Bio’s Tapestri platform

**Fig. 1:**
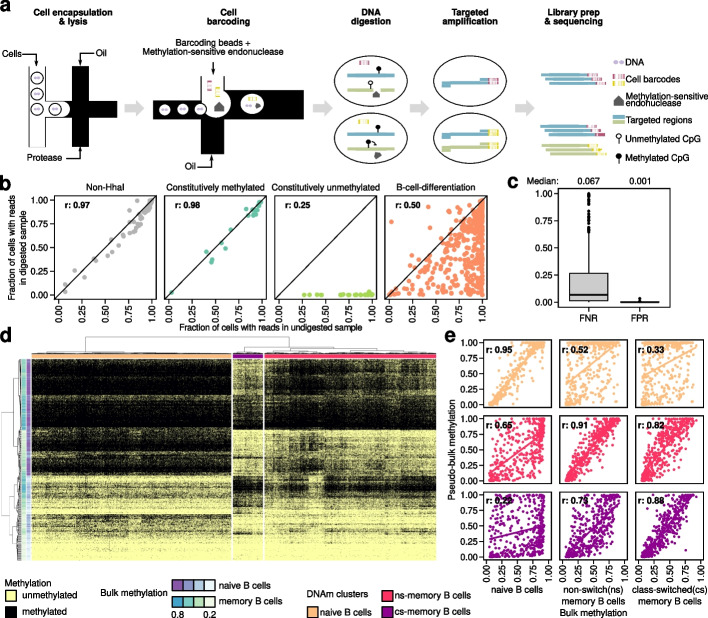
ScTAM-seq identifies cellular subtypes in B cells from peripheral blood. **a** Overview of the scTAM-seq workflow. **b** Per amplicon comparison of fraction of cells with at least one sequencing read in the undigested and digested bone marrow sample. **c** False-negative rate (estimated on B-cell differentiation amplicons, *n*=424, undigested bone marrow control) and false-positive rate (estimated on the constitutively unmethylated amplicons, *n*=32, digested bone marrow sample) across all amplicons of the respective class. **d** Heatmap (binary distance, Ward’s method) of binarized, single-cell DNAm values of 9583 cells across 313 high-performance amplicons in the peripheral blood sample. **e** Comparison of pseudo-bulk and bulk DNAm for the 424 B-cell differentiation-related amplicons. *r*: Pearson’s correlation coefficient. The colors correspond to the DNAm clusters in **d**

We have developed scTAM-seq (single-cell Targeted Analysis of the Methylome) for measuring DNAm states at single-cell resolution in a high-throughput manner. To achieve this, we have combined an optimized single-cell PCR in droplet-based technology (Mission Bio Tapestri platform [17]) with the digestion of genomic DNA using a DNAm-sensitive endonuclease. Among five candidate enzymes, we identified HhaI and SsiI as fully active in the Tapestri Barcoding Mix buffer (see “Methods”) and used HhaI in the following experiments. This enzyme selectively digests unmethylated “GCGC” recognition sites, while leaving methylated sites intact. Hence, upon digestion of genomic DNA in barcoded single-cell droplets, amplicons containing targeted CpGs can only be amplified from methylated recognition sites (Fig. 1a). Based on the throughput of the Tapestri platform, scTAM-seq enables the analysis of 650 CpGs in up to 10,000 cells and can be combined with single-cell readouts of surface-protein expression [18, 19].

For a pilot study, we designed a panel of primers (Additional file 2: Table S1) to amplify 424 amplicons containing CpGs with dynamic DNAm during B-cell differentiation. These include CpGs differentially methylated across hematopoietic stem and progenitor cells (HSCs), pre-B cells, immature B cells, naive B cells, and memory B cells [10]. As controls, we designed amplicons without HhaI recognition sites (non-HhaI), amplicons covering CpGs that are either constitutively methylated or unmethylated across B-cell differentiation, and amplicons interrogating CpGs within imprinting control regions. We applied scTAM-seq to B cells isolated from either bone marrow or peripheral blood, while simultaneously profiling the expression of 46 cell-surface proteins by staining cells with oligonucleotide-tagged antibodies [18, 19]. For both samples, we generated data from two experimental conditions, one digested by HhaI and one undigested sample. Using the per-cell performance of the non-HhaI control amplicons as a quality filter, we obtained data for 4706–9583 cells per experiment (“Methods” and Additional file 3: Table S2).

### scTAM-seq exhibits low false-positive and false-negative rates

To assess the false-negative rate (FNR) and false-positive rate (FPR) of scTAM-seq, we compared the performance of the digested and undigested samples for the bone marrow (Fig. 1b) and blood (Additional file 1: Fig. S2a) samples. Non-HhaI and constitutively methylated amplicons exhibited similar fractions of cells with mapped reads in the undigested and digested samples (e.g., *r* = 0.98, constitutively methylated amplicons from bone marrow samples), showing an amplicon-dependent dropout rate that is virtually identical in the digested and undigested sample (Fig. 1b, Additional file 1: Fig. S2a). Amplicons containing constitutively unmethylated CpGs in B cells only displayed reads in the undigested sample. From these amplicons, we inferred a median FPR (i.e., the number of cells with reads for a given constitutively unmethylated amplicon in the digested sample) of less than 0.2% (Fig. 1c), demonstrating high digestion efficacy.

Most of the amplicons targeting B-cell differentiation CpGs showed a lower fraction of cells with reads in the digested sample, indicating selective digestion of unmethylated CpGs (Fig. 1b). For these amplicons, we leveraged the undigested control sample to calculate the FNR, defined as the number of cells without a sequencing read for a given B-cell differentiation amplicon in the undigested sample. The median FNR was 6.7% for the undigested bone marrow sample and 16.3% for the undigested blood sample, at the level of single cells and single CpGs (Fig. 1c, Additional file 1: Fig. S2b). The larger FNR of the undigested blood sample can be attributed to lower sequencing depth (Additional file 3: Table S2) and an altered distribution of reads per amplicon compared to the undigested bone marrow sample (Additional file 1: Fig. S3).

Finally, considering the non-HhaI and the constitutively methylated amplicons, we observed the highest correlation of both digested samples with the undigested bone marrow sample (Additional file 1: Fig. S4), and exclusively used this sample as control in the following analyses. We note that FNR is influenced by the sequencing depth (“Methods” and Additional file 3: Table S2) and GC content of the amplicon (Additional file 1: Fig. S5). As the FNR can be calculated from the undigested control sample, it can be accounted for when computing pseudo-bulk methylation values in the digested sample (see “Methods”).

### DNAm is dynamic across single cells in peripheral blood

To evaluate the ability of scTAM-seq to resolve cell populations, we first analyzed B cells from the peripheral blood sample. Unsupervised clustering of the CpGs covered by high-confidence amplicons (FNR < 0.25) identified three clusters. We used reference bulk DNAm data to identify these clusters as naive, non-switched (ns-) memory, and class-switched (cs-) memory B cells (Fig. 1d). Surface-protein expression data further validated these cluster assignments and were in line with single-cell CITE-seq data [20] (Additional file 1: Fig. S6 and Fig. S7). We next computed pseudobulk DNAm values per cell-type cluster for all 424 CpGs included in the assay while accounting for the FNR per amplicon (“Methods”). We found a strong correlation between pseudobulk and reference bulk data [10, 21] for each pairwise comparison (*r* ≥ 0.88, Fig. 1e, Additional file 1: Fig. S8), confirming the high accuracy of scTAM-seq.

**Fig. 2:**
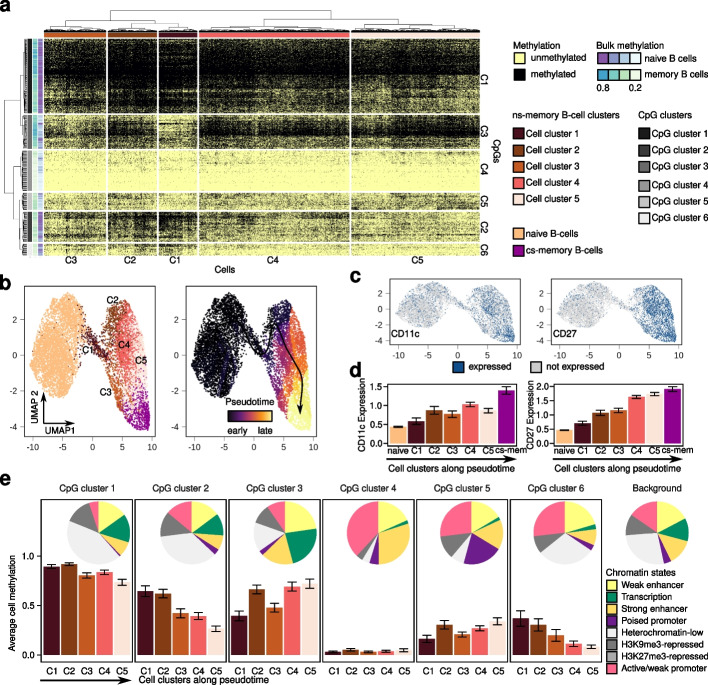
scTAM-seq identifies cellular states associated with proliferation. **a** Heatmap showing the binarized, single-cell DNAm matrix for 4100 ns-memory B cells in the 313 high-performance amplicons. Five clusters of ns-memory B cells and six CpG clusters were defined based on a hierarchical clustering (binary distance, Ward’s method). **b** Low-dimensional representation of the binarized data matrix for all cells (naive, cs- and ns-memory B cells) using UMAP. The pseudotime was inferred with Monocle3. **c** Surface-protein expression within the UMAP-space. The surface-protein expression data was binarized using a cutoff of 1 for the CLR-normalized counts. **d** Surface-protein expression for the different clusters (ordered by increasing pseudotime) identified in **a** as barplots. Shown is the mean and two times the standard error within each of the clusters. **e** Average DNAm value per CpG- and cell cluster were estimated by computing the fraction of all methylated amplicons in a given CpG and cell cluster. The error bar indicates two times the standard error across all cells of a cell cluster. The pie chart indicates the genomic distribution of the CpGs within each CpG cluster according to chromatin states of naive, germinal center, ns-, and cs-memory B cells defined in Beekman et al.

To assess if scTAM-seq can resolve intra-population heterogeneity, we further performed a focused analysis of the 4100 ns-memory B cells. We identified substantial heterogeneity within this population at the DNAm level, which was linked to differences in the expression of CD27 and CD11c cell-surface proteins (Fig. 2a–d). The gradual gain of CD27 appeared to be associated with gradual progression of cellular differentiation, with CD27-negative ns-memory B cells representing early, atypical memory B cells that are present at the highest frequencies at birth [22, 23]. Furthermore, linking the different DNAm patterns within the ns-memory B cell cluster to chromatin states [24] showed that one CpG cluster that loses methylation along differentiation pseudotime (CpG cluster 1) is enriched for heterochromatin (Fisher’s test *p*-value: 2.28×10^−9^), while gain of methylation (CpG cluster 5) occurs more frequently in polycomb-associated poised promoters (Fisher’s test *p*-value: 2.7×10^−4^) (Fig. 2e). These patterns have previously been linked to proliferative history [25], showing that scTAM-seq can capture the gradual increase in proliferative history upon cellular differentiation. Additionally, subsets of memory B cells expressing CD11c have previously been described as more prone towards differentiation into plasma cells and are enriched in autoimmune diseases [26, 27]. Overall, our results show the potential of our method to resolve cell types to an unprecedented resolution, characterizing subpopulations masked until now within bulk DNAm data, and linking them to other mechanisms such as gradual differentiation and proliferative history.

### Surface-protein expression data can be leveraged for integration of high-throughput scDNAm data with proteo-transcriptomic atlases

**Fig. 3:**
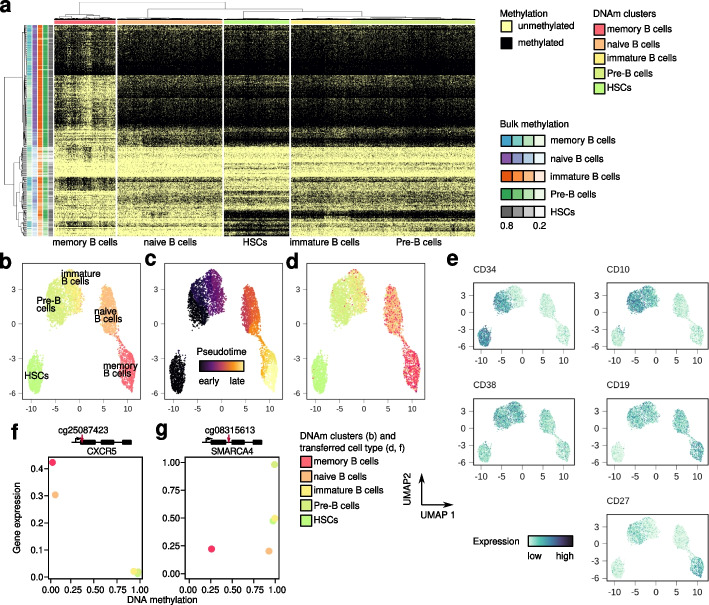
scTAM-seq captures the B-cell differentiation process in bone marrow. **a** Heatmap of single-cell DNAm values for 5340 cells and 313 amplicons in the digested bone marrow sample. **b–d** Visualization of DNAm data in low-dimensional space (UMAP), with labels inferred from bulk DNAm (**b**), across differentiation pseudotime (inferred with Monocle3, **c**), or with labels transferred from CITE-seq data (**d**). **e** Visualization of surface-protein expression for cells embedded in the DNAm UMAP. The value shows the CLR-normalized expression values (see “Methods”). **f**, **g** Relationship between DNAm and log-normalized gene expression across B-cell differentiation showing a positive (**f**, CXCR5, CpG located in promoter region) and negative correlation (**g**, SMARCA4, CpG located in intronic region), respectively

We next investigated the potential of scTAM-seq to profile DNAm dynamics during B-cell differentiation in bone marrow. Dimensionality reduction and clustering using scDNAm data revealed five groups of cells arranged in a putative B-cell differentiation pseudotime trajectory [28]. We labelled the clusters as hematopoietic stem and progenitor cells (HSCs), pro/pre-B cells (pre-B cells), immature B cells, naive B cells, and memory B cells based on pseudotime and reference bulk DNAm patterns (Fig. 3a–c, Additional file 1: Fig. S9-S11). Notably, naive, cs-, and ns-memory B cells were represented both in the bone marrow and the blood samples and highlighted a high correlation between pseudo-bulks of biological replicates analyzed with scTAM-seq (all correlations *r*> 0.92, Additional file 1: Fig. S12).

To identify cell types in an automated manner without making use of bulk DNAm levels, we transferred labels from a single-cell CITE-seq reference [20], using the surface-protein expression data captured by scTAM-seq (Fig. 3d). These annotations, as well as the expression of cluster-specific cell-surface proteins (Fig. 3e), confirmed the order of B-cell differentiation stages by pseudotime analysis. Next, we demonstrated that the surface-protein expression data can be used to integrate scDNAm and scRNA-seq data into a common reference space (Additional file 1: Fig. S13). This allowed us to identify CpGs anti-correlated and correlated with gene expression throughout the differentiation trajectory, as exemplified for CXCR5 (negative correlation, CpG located in gene promoter) and SMARCA4 (positive correlation, CpG located in intronic region, Fig. 3f,g). Together, these analyses demonstrate the abilities of scTAM-seq to dissect DNAm heterogeneity of complex cellular populations.

## Discussion

We present scTAM-seq as a targeted, cost-effective, scDNAm method with an FPR of less than 0.2% and an FNR as low as 7%, depending on the amplicon GC content and sequencing depth. In silico simulations show that the FPR and FNR of scTAM-seq estimated from the digested and undigested bone marrow and blood samples are suited to reliably cluster cells and to estimate pseudo-bulk DNAm values (Additional file 1: Fig. S14). As scTAM-seq is a targeted method, only up to 650 CpGs of the 28 million CpGs in the human genome can be analyzed. This might lead to a decreased ability for de novo cell-type discovery in comparison to large-scale projects employing genome-wide scDNAm methods [29]. On the other hand, genome-wide approaches for scDNAm profiling suffer from limited cellular throughput and high data sparsity. Targeted methods thus serve as complementary approaches to genome-wide (single-cell) DNAm profiling methods, since the reduction in data sparsity allows for a more detailed analysis of selected CpGs in a large number of cells, rather than having to bin single-cell methylation states across genomic windows. Additionally, for most tissues and cell types, bulk DNAm data is readily available, revealing that only few CpGs are variable across cell differentiation (Additional file 1: Fig. S1). Thus, targeting only a few, highly variable CpGs is promising to reveal DNAm heterogeneity across cellular differentiation at unprecedented accuracy. Of note, the Mission Bio Tapestri platform has recently been extended to cover 1000 amplicons, which increases the coverage of scTAM-seq even further. The selection of potentially interesting CpGs for scTAM-seq is facilitated by tools for the identification of variably methylated sites from bulk DNAm data [12]. We created a pipeline automating the selection of such target sites for scTAM-seq, which is available at https://github.com/veltenlab/CpGSelectionPipeline [30]. Of note, the use of HhaI limits the number of analyzable CpGs to 1.76 million in the human genome, but further sites can be analyzed using other enzymes such as SsiI (Additional file 4: Table S3).

Since scTAM-seq exhibits a low FNR and FPR, we envision that it can also be used to further investigate imprinted regions, as well as other regions harbouring allele- and strand-specific methylation (Additional file 1: Fig. S15). Ultimately, scDNAm values can help to discern cellular heterogeneity from allele-specific methylation, which in bulk data can only be achieved in special situations where SNPs are located on the same sequencing read. Conversely, allele- and strand-specific methylation might lead to an overestimation of pseudo-bulk DNAm values by scTAM-seq.

Of note, the Mission Bio Tapestri platform was originally developed for profiling somatic mutations in cancer at single-cell resolution [18]. This ability can readily be integrated into scTAM-seq. Sites of potential somatic mutations can regularly be included into the amplicon panel design and thus profiled together with the cell-type-specific DNAm state (as long as there is no HhaI cut site in the amplicon). Thereby, scTAM-seq has the potential to uncover clonal and sub-clonal development of tumors from the combined angle of somatic mutation, DNAm, and cell-surface proteins. Furthermore, it allows us to characterize surface-protein phenotypes of cells defined by specific DNAm patterns and to develop FACS schemes for purifying these newly identified cellular identities [20]. To guide cell-type annotation, the surface-protein expression data can be used for integration with proteo-transcriptomic atlases, as exemplified for the bone marrow sample. Finally, in addition to human panels, murine DNAm dynamics can also be profiled.

## Conclusions

In summary, scTAM-seq is a powerful method for investigating DNAm dynamics at single-cell and single-nucleotide resolution. Due to its targeted nature, it focuses sequencing coverage on CpGs with variable methylation states in a cell population of interest, achieving a false-positive rate of less than 0.2% combined with a false-negative rate as low as 7%. Therefore, scTAM-seq alleviates data sparsity in comparison to genome-wide methods and thus enables high-throughput cell-to-cell comparisons at the level of single CpGs.

## Methods

### Amplicon panel design

#### Enzyme selection

Using the REBASE database (http://rebase.neb.com/rebase/rebase.html [31]), 60 DNAm-sensitive endonucleases, targeting 33 restriction sites and fulfilling the following criteria, were selected: (i) digestion activity is completely blocked by DNAm (5mC), (ii) enzyme is sensitive to heat inactivation, (iii) recognition site does not contain Ns (representing any nucleotide), (iv) recognition site harbors a single CpG, (v) enzyme is commercially available. The complete selection of DNAm-sensitive endonucleases is available in Additional file 4: Table S3. Next, we chose four enzymes to test in the Tapestri Barcoding Mix buffer: AciI (NEB, recognition site CCGC), HpaII (NEB, recognition site CCGG), HpyCH4IV (NEB, recognition site ACGT), and HhaI (NEB, recognition site GCGC), with 3.7 M, 2.3 M, 2.2 M, and 1.3 M recognition sites in the human genome (hg38), respectively. To comply with criterium (iv), sequence contexts in which two CpGs occur, e.g., CCGCG for AciI and, CGCGC or GCGCG for HhaI, are not included within this list; but without this consideration, there are 4.2 M AciI, and 1.76 M HhaI recognition sites in the human genome. These numbers were calculated using Biostrings v2.56.0, R package (https://bioconductor.org/packages/Biostrings).

The activity of these four enzymes was tested as follows. PCR products were generated from genomic DNA of Jurkat cells using the primers: 5′-TTCCACGTTTTTCTTTCATGC-3′ and 5′-GCAGTCGTTGGTTGGAAACT-3′for AciI, 5′-CCCAGGCGTTTGTTAAAGAG-3′and 5′-GCATGAAAGAAAAACGTGGAA-3′ for HpaII, 5′-TGGCTGTAGCCAGTTCTCAA-3′ and 5′-AAGGACACGCCTCTCACACT-3′ for HpyCH4IV, 5′-GGGGATCAATCACCATATGAA-3′and 5′-TGGCTGATGGGATCAACAAT-3′ for HhaI, followed by digestion of the respective amplicons for 30 min at 37°C in the standard buffer provided by the company, or the Tapestri Barcoding Mix buffer. HpaII and HpyCH4IV showed only partial digestion in the Tapestri Barcoding Mix buffer. On the contrary, HhaI showed the same ability to digest unmethylated PCR products in both buffers (data not shown) and was chosen for the follow-up scTAM-seq design. AciI did not show complete digestion in the Tapestri Barcoding Mix buffer, but its isoschizomer SsiI (Thermo Fisher) showed almost complete digestion, comparable with the standard buffer (data not shown), representing an alternative enzyme compatible with scTAM-seq.

#### Target region selection

Target regions were selected fulfilling the following criteria: (i) containing one HhaI recognition site in a 300-bp window (except for non-HhaI amplicons, which do not contain any recognition site), (ii) GC content between 45 and 65%. The first criterion reduces the number of CpGs that can be analyzed by SsiI (scTAM-seq compatible) and HhaI to 1.6M and 0.8M, respectively. Bulk DNAm data of B-cell subpopulations (stem and progenitor cells, pre-BI and pre-BII cells, immature, naive, non-class-switched and class-switched memory, plasmablasts, and plasma cells) obtained by 450K array analysis (Illumina) were mined from Kulis et al. [10]. Using this data, we identified 1753 CpGs showing dynamic DNAm during B-cell differentiation that can be digested by HhaI and fulfill the criteria above. Next, to select around 450 CpGs with the highest predictive value for the B-cell populations of interest, scDNAm datasets were simulated using the bulk DNAm values of the 1753 CpGs (considering an allelic FNR of 0.2 and an allelic FPR of 0.1). This data was employed to run a regularized linear model (glmnet R package [32], parameters: *λ* = *e*^−4^, *α* = 0.9), leading to the selection of a panel of 428 CpGs. Furthermore, to improve the limited separation between early B-cell subpopulations, 32 CpGs showing differential methylation among these populations (pairwise comparisons, FDR <0.05, minimum DNAm difference 0.25) were added to the panel, yielding a final selection of 451 CpGs. As controls, we selected 50 constitutively unmethylated CpGs within an HhaI recognition site (top 50 CpGs with lowest DNAm levels in B cells, bulk DNAm <0.06 in all samples) and 30 constitutively methylated CpGs within an HhaI recognition site (top 30 CpGs with highest methylation levels in B cells, bulk DNA methylation >0.94 in all samples), as well as 96 amplicons without HhaI recognition sites. Moreover, we leveraged imprinting control region data from Court et al. [33] to select 13 CpGs showing intermediate methylation across B-cell differentiation.

#### Amplicon design

Using the Tapestri Designer tool (https://support.missionbio.com/hc/en-us/articles/4404329631895-Tapestri-Designer-User-Guide), amplicons were designed spanning our CpGs of interest. Some regions yielded no amplicons, leading to a final panel covering 424 B-cell differentiation-related CpGs, 21 constitutively methylated CpGs, 32 constitutively unmethylated CpGs, 12 CpGs within imprinting control regions, and 87 non-HhaI regions. A complete overview of the panel design can be found in Additional file 2: Table S1.

#### Recommendations for panel design

Since amplicon performance is related to GC content, we recommend excluding amplicons with a GC content above 0.65 (Additional file 1: Fig. S5). Non-HhaI amplicons are essential to distinguish between cells and empty droplets after sequencing, we recommend including at least 50 amplicons of this type in the design. To aid the inclusion of these control regions in other panels, we provide the exact locations of the high-confidence non-HhaI amplicons used for cell selection in our study in Additional file 5: Table S4.

### Sample preparation

#### Sample description

Peripheral blood samples were obtained from two healthy donors (female age 57, male age 49) from the *Banc de Sang I Teixits* (Catalunya, Spain). Frozen bone marrow mononuclear cells were purchased from StemExpress® (Folsom, CA, USA, Cat. num. BMMNC050C; Lot. 2106150113) and correspond to a 22-year-old healthy male donor.

#### Isolation of B-cell subpopulations from peripheral blood

Briefly, peripheral blood was collected and stored at room temperature (15–25°C). Within 24 h after sample collection, B-cell subpopulations were isolated using the RosetteSeqTM Human B Cell Enrichment Cocktail (StemCellTM Technologies; Cat. num. 15024) followed by a Ficoll®-Paque Premium (Gmbh; Cat. num. 17-5442-02) density gradient centrifugation. B-cell subpopulations were cryopreserved in heat-inactivated fetal bovine serum (GibcoTM; Cat. num. 10270106) supplemented with 10% DMSO, until the day of the experiment.

#### Flow cytometry cell sorting

B-cell subpopulations from PBMCs were stained with the monoclonal antibody anti-CD19(PE) (Clone HIB19; Invitrogen; Cat. num. 12-0199-41) in a 1:20 dilution for 30 min on ice. Bone marrow mononuclear cells were stained for 30 min on ice with the following antibodies: anti-CD19(APC/Cy7) (clone HIB19; Biolegend; Cat. num. 302217) in a 1:20 dilution, anti-CD38(APC) (clone HIT2; eBioscience; Cat. num. 17-0389-42) in a 1:30 dilution, anti-CD123(PE) (clone 763; BD; Cat. num. 561058) in a 1:50 dilution, anti-CD10(PE/Cy7) (clone Hi10a; Biolegend; Cat. num. 312213) in a dilution 1:20, and anti-CD34(AF488) (clone 581; Biolegend; Cat. num. 343517) in a dilution 1:100. All samples were sorted using the BD Influx and FACSAria II SORP cell sorters. A purity of 90–95% CD19+ cells was obtained after sorting PBMC samples. The bone marrow sample was sorted for the following subpopulations: (S1) CD34+/CD38-, (S2) CD38+/CD34+/CD10+/CD123- and (S3) CD19+/CD34-, resulting in 9.25% of S1, 6.5% of S2 and 84.25% of S3 when considering the total number of sorted cells. These subpopulations were mixed in a ratio of 10% of S1, 22.9% of S2, and 67.1% of S3 for a total of 1 M cells.

#### Oligo-tagged antibody cell staining

Following sorting, cells were stained with oligo-tagged antibodies following the instruction in the Tapestri® Single-Cell DNA + Protein Sequencing User Guide V2 (Tapestri User Guide V2, https://support.missionbio.com/hc/en-us/articles/360062406493-Tapestri-Single-cell-DNA-Protein-Sequencing-V2-User-Guide). Briefly, 1 M cells were stained with TotalSeq-D Heme Oncology Cocktail (BioLegend; Cat. num. 399906) reconstituted in 59 μl of Cell Staining buffer (BioLegend; Cat. num. 420201), and adding 1 μl of TotalSeq-D0154 anti-CD27 antibody (clone O323, BioLegend; Cat. num. 302861) for 30 min on ice.

### scTAM-seq and surface protein library preparation and sequencing

#### Sample processing using the Tapestri instrument

A total of 120,000–140,000 cells were loaded into a Tapestri microfluidics cartridge. Upon encapsulation, cells were lysed. To obtain the digested samples, the DNAm-sensitive endonuclease was added to the barcoding master mix as follows: 288 μl of Tapestri Barcoding Mix V2, 5 μl of highly concentrated HhaI enzyme (150,000 U/mL, NEB; Cat. Num. R0139B-HC1), while keeping the remaining reagents as stated in the Tapestri protocol (5 μl Forward Primer Pool, 2 μl Antibody Tag Primer). To ensure the activity of the DNAm-sensitive endonuclease, the PCR program was modified as shown in Additional file 6: Table S5, introducing a 30-min step at 37°C, prior to the targeted amplification. This step was introduced for the digested and undigested samples to avoid bias in the amplification performance. The DNA digestion and targeted PCR steps were performed in a T100 Thermal Cycler (Bio-Rad). All other sample processing steps were performed following the Tapestri User Guide V2. All Tapestri-related reagents were obtained using Tapestri Single-Cell DNA Custom Kits and Cartridge (Mission Bio, Inc; Cat. num. MB02-0001 and MB03-0034).

#### Recommendations for sample preparation

The scTAM-seq protocol has been optimized utilizing a highly concentrated HhaI enzyme (150,000 U/mL, NEB; Cat. Num. R0139B-HC1). To ensure the stability of the single-cell emulsion, we recommend using this highly concentrated enzyme. Regarding the undigested control sample, we suggest generating at least one undigested control sample per amplicon panel. To ensure this sample is of sufficient quality, we suggest to evaluate the correlation of the non-HhaI amplicons between the undigested sample and each digested sample, as shown in Additional file 1: Fig. S4. If more than one undigested sample is generated, we suggest to use as a control the undigested sample that shows the highest correlation with each digested sample, respectively.

#### scTAM-seq and surface-protein library purification and sequencing

The Tapestri User Guide V2 was followed to prepare the libraries, with a few modifications listed here. Briefly, PCR products were retrieved from individual droplets and purified with 0.7X Ampure XP beads (Beckman Coulter; Cat. num. A63881), to split the scTAM-seq library bound to the beads from the surface-protein library in the supernatant. Illumina i5/i7 sequencing indexes were added to the scTAM-seq PCR products, followed by two steps of purification, using 0.69X and 0.65X Ampure XP beads (these are different from the ratios indicated in the Tapestri User Guide V2). PCR products from the surface-protein library were incubated with Tapestri Biotin Oligo at 96 °C for 5 min, followed by incubation on ice for 5 min, and purified using Tapestri Streptavidin beads. Afterwards, the beads were used as PCR templates for the incorporation of i5/i7 Illumina indices, followed by purification using 0.9X Ampure XP beads. The quality of all scTAM-seq and surface-protein libraries were assessed by Bioanalyzer (Additional file 1: Fig. S16). Libraries were pooled and sequenced on the Illumina NovaSeq 6000 platform, at the CNAG-CRG Sequencing Unit, read length 2 × 150 cycles, at a sequencing depth of 260 M reads/library in case of surface-protein libraries, and 420 M aligned reads/library for scTAM-seq libraries (Additional file 3: Table S2).

#### Recommendations for sample sequencing

The FNR is related to the sequencing depth. We recommend adhering to the following formula for calculating the number of reads required for scTAM-seq libraries:

- Recommended coverage for each amplicon per cell: 60–80X (preferably closer to the higher end)
- Total paired reads per library: *number of expected cells* × *number of amplicons in the panel* × *coverage per amplicon.*

The number of expected cells per default is 10,000. Therefore, for a panel of 500 amplicons sequenced at 70X, the required paired-end read number of would be (10,000 × 500 × 70) 350M. Moreover, it is important to ensure similar sequence depth per library is achieved among all samples, with the undigested control having the same or more reads per library than the digested sample.

For the surface-protein library, we followed the instructions on Tapestri User Guide V2.

### Bioinformatic analysis

#### Raw data processing

Raw sequencing data was processed using a customized version of the Mission Bio Tapestri Pipeline v2 (https://support.missionbio.com/hc/en-us/sections/360006255314-Tapestri-Pipeline). Briefly, cell barcodes were extracted from the raw FASTQ data files and sequencing adapters trimmed using cutadapt [34] v2.5. Trimmed reads were aligned to the reference genome version “hg19” using bwa-mem [35] v0.7.12. Subsequently, read pair information was verified using PicardTools (v1.126, https://github.com/broadinstitute/picard), and quantified with samtools [36] (v1.9), and the cell barcode distribution was computed using the python scripts provided by Mission Bio. As a final step, we identified cells from the barcodes as follows: we exclusively used the non-HhaI control amplicons to determine barcodes that can reliably be called as “cells” according to their overall read counts and adapted the original cell detection method implemented by Mission Bio (https://support.missionbio.com/hc/en-us/articles/360042381634-Cell-calling). We only used those control amplicons that were reliably captured in most of the cells (Additional file 5: Table S4). To determine a read count cutoff on a per amplicon and per cell basis, we focused on the number of amplicons in the panel and computed the threshold as the minimum of 10 and 0.2 times the average number of reads for those cells covered by at least eight times the number of amplicons in the panel (see https://support.missionbio.com/hc/en-us/articles/360042381634-Cell-calling for a more detailed description). Then, we call as cells those barcodes having more than the determined threshold of reads in at least 70% of the amplicons. To determine potential doublets, we employed the DoubletDetection (10.5281/zenodo.2678041) software, which removed a maximum of 16% of cells from our data (Additional file 7: Table S6, Additional file 1: Fig. 17). The pipeline has been implemented in *bash* and *snakemake* [37] and is available from the GitHub repository (https://github.com/veltenlab/scTAM-seq-scripts) [38].

#### Clustering analysis and dimension reduction

As a first step, we computed false-positive rates (FPR) and false-negative rates (FNR) as follows: the FPR represents the event when a read is present although the respective site is not methylated; therefore, we leveraged the 32 amplicons containing constitutively unmethylated CpGs in the digested sample to compute a FPR for each amplicon across all the cells. The FPR is defined as the fraction of cells that obtain at least one sequencing read across all cells for the constitutively unmethylated amplicons. Conversely, the FNR represents the probability of observing no sequencing read although there is either no digestion or the site is protected by methylation. We were particularly interested in the FNR of the 424 B-cell differentiation amplicons and thus quantified their FNR in the undigested sample. Consequently, we computed the per amplicon FNR as the fraction of cells not having a sequencing read across all cells.

Next, we discretized the cells-by-amplicon DNAm matrix according to the presence of at least one sequencing read and clustered the binary matrix to obtain cell-type clusters. Due to the low FPR computed in the control experiment, we found that a cutoff of one sequencing read reliably differentiated methylated from unmethylated CpGs for an individual cell. We selected high-performance amplicons as those amplicons with FNR lower than 0.25 in the undigested bone marrow control, leading to 313 amplicons selected. Notably, a fraction of cells in the amplicons could be false positives, but we found that this effect is diminished by cell clustering and pseudo-bulk computation. We clustered this matrix using the binary distance (i.e., the fraction of methylation calls that are different between any two cells divided by the fully methylated states) and Ward’s minimum variance method (“ward.D2” option in the “hclust” R function). We selected three clusters for the peripheral blood and five clusters for the bone marrow data, since we observed a separation into previously defined cell types.

To obtain a low-dimensional representation of the data, we used the read count matrix obtained from scTAM-seq after removal of doublets, and selected the 313 high-performance amplicons associated with B-cell differentiation. After binarization as stated above, we normalized the data using Seurat v4.0 [39] with the functions “NormalizeData” (“LogNormalize” method), and then executed “ScaleData,” “RunPCA,” “FindNeighbors” (dimensions 1–11), and “RunUMAP” using all of the features. The cells of the bone marrow sample were annotated using the information from bulk data into five cell types. Then, we used Signac (v1.2) [40] and Monocle3 (v1.0) [28] to infer a trajectory in the low-dimensional space (“learn_graph” and “order_cell” functions).

For the surface protein expression data, we used Seurat v4.0 and employed the centered log ratio (CLR) method for normalization. All surface-protein expression values shown in the paper represent the CLR-normalized expression values.

#### Computing pseudo-bulk DNAm values

We aimed at obtaining a pseudobulk DNAm level across all cells in a given cluster on a per CpG basis, correcting for the FNR observed in the undigested sample. Notably, we aimed at inferring pseudobulk DNAm values for all 424 B-cell differentiation amplicons, also for those with elevated FNRs. We defined the observables for amplicon *i* and cell cluster *c*, which can be computed directly from the experiments as follows:

$${n}_{0,i,c}=\textrm{Number}\ \textrm{of}\ \textrm{cells}\ \textrm{without}\ \textrm{any}\ \textrm{read}\ \textrm{for}\ \textrm{amplicon}\ i\ \textrm{in}\ \textrm{cluster}\ c$$

$${n}_{1,i,c}=\textrm{Number}\ \textrm{of}\ \textrm{cells}\ \textrm{with}\ \textrm{at}\ \textrm{least}\ \textrm{one}\ \textrm{read}\ \textrm{for}\ \textrm{amplicon}\ i\ \textrm{in}\ \textrm{cluster}\ c$$

$${p}_i=\textrm{FNR}\ \textrm{for}\ \textrm{amplicon}\ i\ \textrm{estimated}\ \textrm{from}\ \textrm{the}\ \textrm{undigested}\ \textrm{control}\ \textrm{experiment}$$

The parameter we aim to estimate is the DNA methylation value for cluster *c* in amplicon *i*:

$${m}_{i,c}=\textrm{Pseudobulk}\ \textrm{methylation}\ \textrm{state}\ \textrm{of}\ \textrm{amplicon}\ i\ \textrm{in}\ \textrm{cluster}\ c$$

We assume that the reads that we obtained can be modelled using a Binomial distribution given the following formula:

$${n}_{1,i,c}\sim Binom\left({n}_{0,i,c}+{n}_{1,i,c},\left(1-{p}_i\right)\times {m}_{i,c}\right)$$

Additionally, we assume that we do not have prior information about the true underlying DNA methylation state and thus use an uninformative prior:

$${m}_{i,c}\sim Beta\left(1,1\right)$$

We use the Hamiltonian Monte Carlo algorithm as implemented in the “rstan” R package [41] to obtain the parameter *m*_*i,c*_ (pseudobulk methylation value) for each cluster separately.

#### Integrating scTAM-seq with scRNA-seq data

Since 33 of the 46 surface proteins were also measured in a single-cell proteo-transcriptomic atlas [20], we used this overlap to perform cell label transfer and data integration of the bone marrow data with the CITE-seq reference atlas [20] using Seurat v4.0 [39]. Multimodal nearest neighbors are defined between cells assayed using scTAM-seq and CITE-seq data using the joint information of surface-protein expression with the “FindMultiModalNeighbors” function. Then, we used the “RunSPCA” function to perform supervised PCA (sPCA) of the surface-protein expression data in the reference atlas and determined anchors in our dataset using the “FindTransferAnchors” function. Lastly, we used the “MapQuery” function of Seurat to transfer the cell-type label from the reference atlas to our dataset. For this task, we summarized pre-, pro-, and pre-pro-B cells into one class which we termed pre-B cells. After joining the reference atlas and our data using the sPCA dimension reduction, we generated a new low-dimensional representation of the combined dataset using the “RunUMAP” function.

For associating DNAm differences with gene expression changes, we leveraged the labels transferred from the CITE-seq atlas and correlated the mean expression from the whole transcriptome analysis of Triana et al. [20] with the mean DNAm per cell type for the five cell-type labels transferred. Importantly, we only considered those genes that are located closer than 25kb in both orientations from the investigated CpG. Then, we investigated those genes showing the strongest (negative/positive) Pearson correlation with the target CpG. We found that there were more negatively correlated than positively correlated genes and that the CpGs with the strongest correlation were preferentially located in the gene promoter (Additional file 8: Table S7 and Table S8).

#### Data visualization

All analyses were performed with R-version newer than 4.0 [42] and the ggplot2 (https://ggplot2.tidyverse.org) and ComplexHeatmap [43] R packages were used for plotting. The lines in the boxplot represent the median, the 25th and 75th percentiles, and 1.5 times the inter-quartile range.

## Supplementary Information

Additional file 1. Supplementary Figures S1-S17.

Additional file 2: Table S1. Design overview of the panel of targeted regions.

Additional file 3: Table S2. Sequencing details per sample and condition.

Additional file 4: Table S3. Complete list of CpG methylation-sensitive endonucleases potentially compatible with scTAM-seq.

Additional file 5: Table S4. Non-HhaI amplicons used for cell selection.

Additional file 6: Table S5. PCR program for DNA digestion and targeted amplification steps.

Additional file 7: Table S6. Doublet detection details per sample and condition.

Additional file 8: Table S7 and Table S8. Correlation analysis between target CpGs and gene expression.

Additional file 9. Review history.

## Data Availability

Raw and processed DNA methylation and surface-protein expression data is available from the Gene Expression Omnibus (GEO) under accession number GSE198019 [44]. All associated code to reproduce the analysis is available under the MIT license (https://opensource.org/licenses/MIT) from https://github.com/veltenlab/scTAM-seq-scripts [38] and Zenodo: 10.5281/zenodo.7233797 [45]. Additionally, a pipeline for selecting CpGs for scTAM-seq is available from https://github.com/veltenlab/CpGSelectionPipeline [30].
