# Supplementary material for: scTAM-seq enables targeted high-confidence analysis of DNA methylation in single cells
Source: Genome Biol. 2022 Oct 28;23:229. doi: 10.1186/s13059-022-02796-7 (PMC9615163; doi:10.1186/s13059-022-02796-7)
Supplement: Supplementary file 1 — Additional file 1. Supplementary Figures S1-S17. [file 13059_2022_2796_MOESM1_ESM.docx]

**Supplementary Figures for “scTAM-seq enables targeted high-confidence analysis of DNA methylation in single cells”**


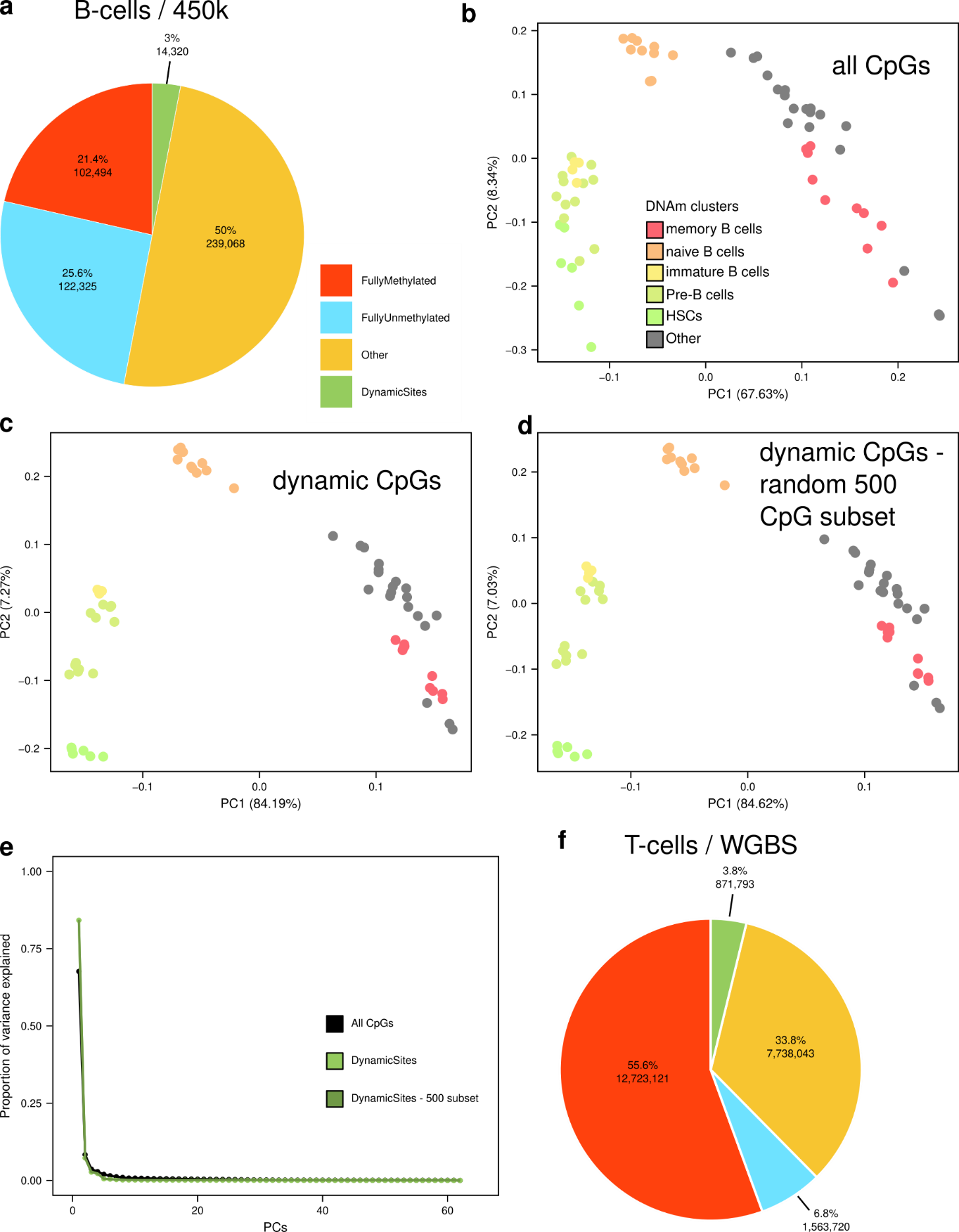


**Fig S1: CpGs dynamically methylated across B- and T-cell development.** **a:** Pie chart estimating the number of dynamic CpGs in human B cells (450k data, Kulis *et al.,* 2015). We used all the replicates of the different cell types (HSCs, preB, immature, naive, and memory B cells). Fully methylated CpGs are defined as CpGs with methylation values higher than 0.75 in all samples and fully unmethylated are those with less than 0.25. We defined as dynamic sites CpGs showing a DNA methylation difference of at least 0.5 in at least one of the pairwise comparisons between the cell types. All remaining CpGs are classified as ‘Others’. PCA for all (478,207) CpGs (**b**), only for the dynamic CpGs (**c**), and for 500 CpGs randomly selected from the set of dynamic CpGs (**d**). **e:** Proportion of variance explained for the PCs shown in **b, c** and **d**. **f:** Analysis similar to **a** for human T cells (naive, central-memory and effector-memory T cells, Durek *et al.,* 2016). DNAm data was generated using WGBS and we focused only on those CpGs covered by at least 10 sequencing reads in all the samples.


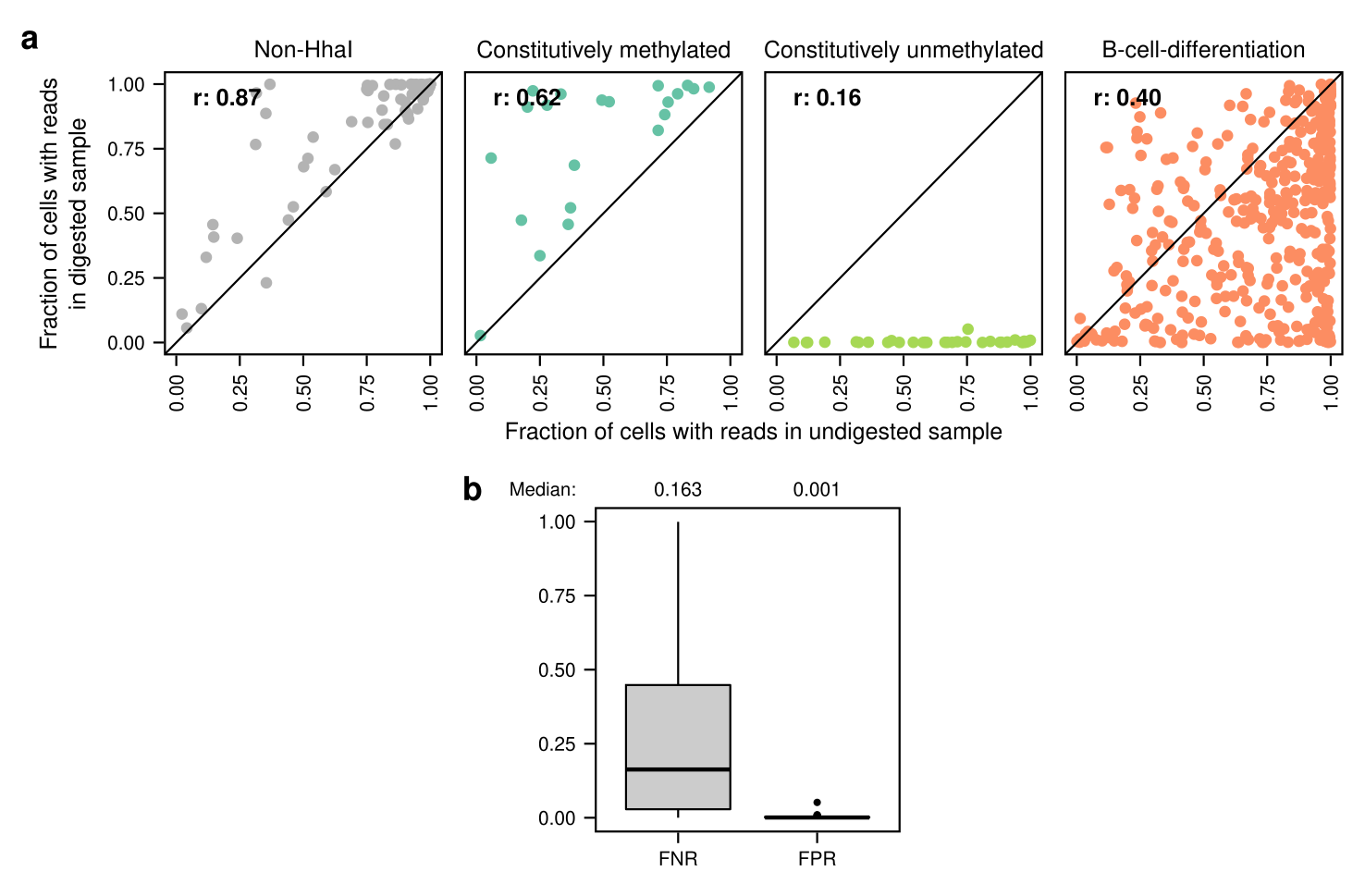
**Fig S2: Estimation of FNR and FPR in the peripheral blood sample.** **a**: Per amplicon comparison of the fraction of cells with at least one sequencing read in the undigested and digested peripheral blood sample for each class of amplicons. **b**: Boxplots and median values for FNR and FPR. The FNR is estimated in the undigested control sample as the fraction of cells that do not receive a sequencing read across the B-cell-differentiation amplicons (n=424). The FPR is defined in the digested sample as the fraction of cells that receive a sequencing read in the always unmethylated amplicons (n=32).


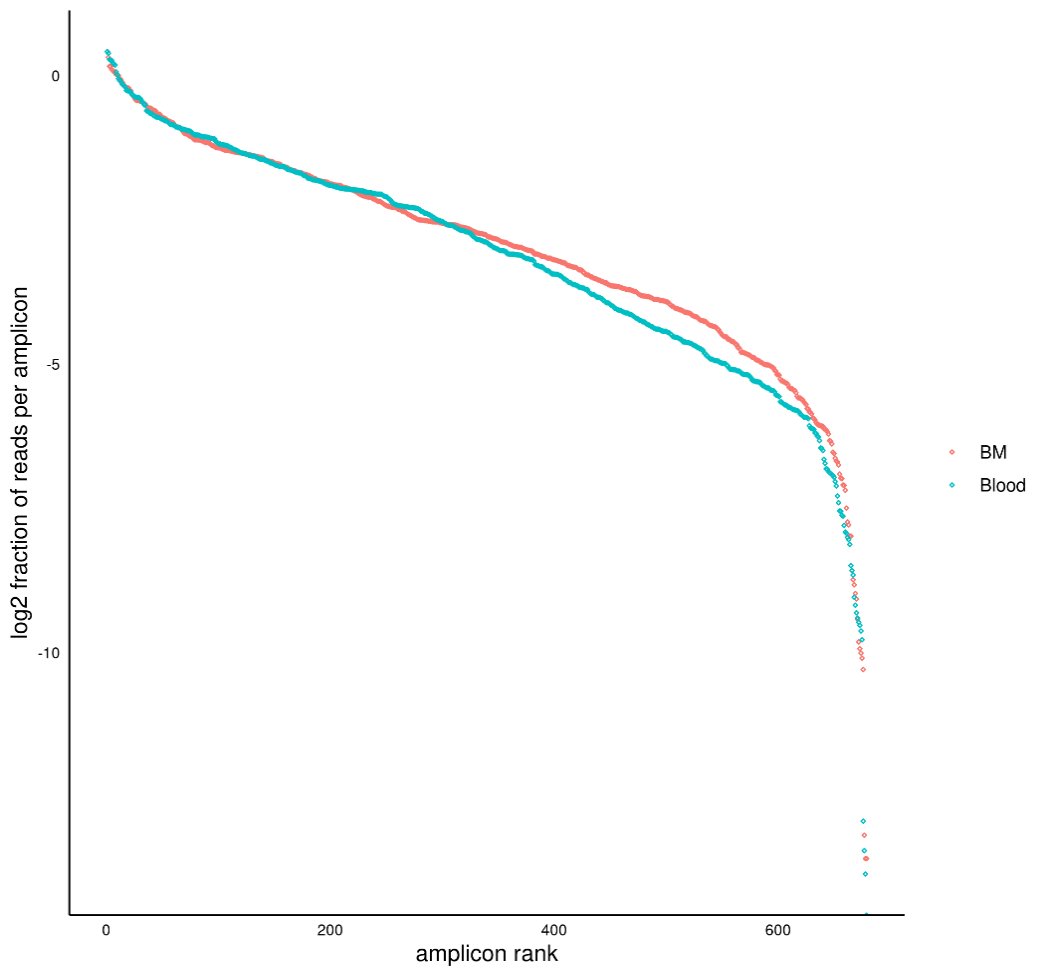

**Fig S3: Comparison of the performance of the undigested samples.** The y-axis shows the fraction of reads per amplicon in the undigested samples (blood/bone marrow). For each sample, amplicons are ranked by the fraction of total reads per amplicon. BM: Bone marrow undigested sample. Blood: Peripheral blood undigested sample.


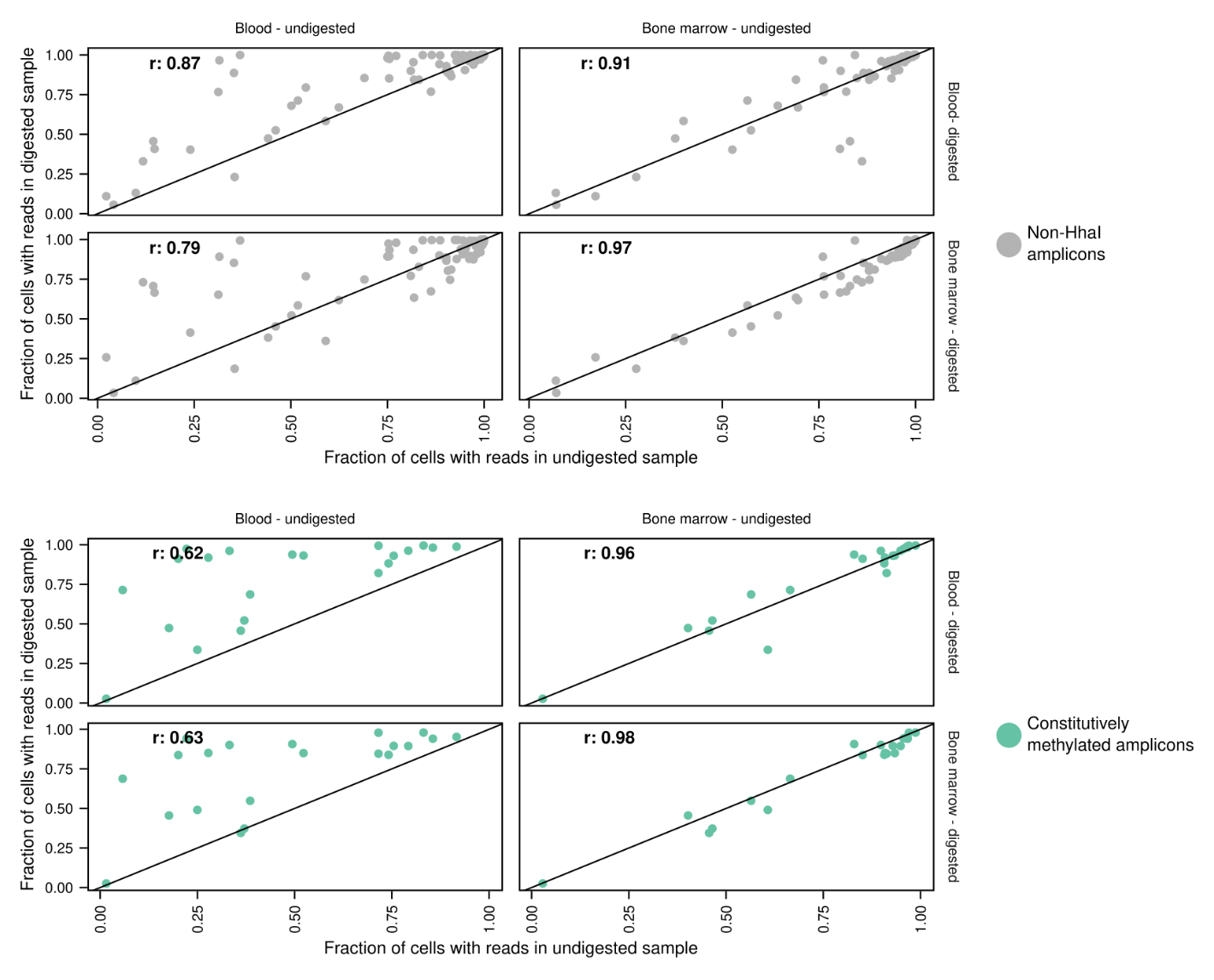
**Fig S4: Comparison of the performance of the digested and undigested samples.** Comparison of the fraction of cells with at least one sequencing read for all digested and undigested samples exclusively for the non-HhaI (n=87) and constitutively methylated (n=21) amplicons. r: Pearson’s correlation coefficient.


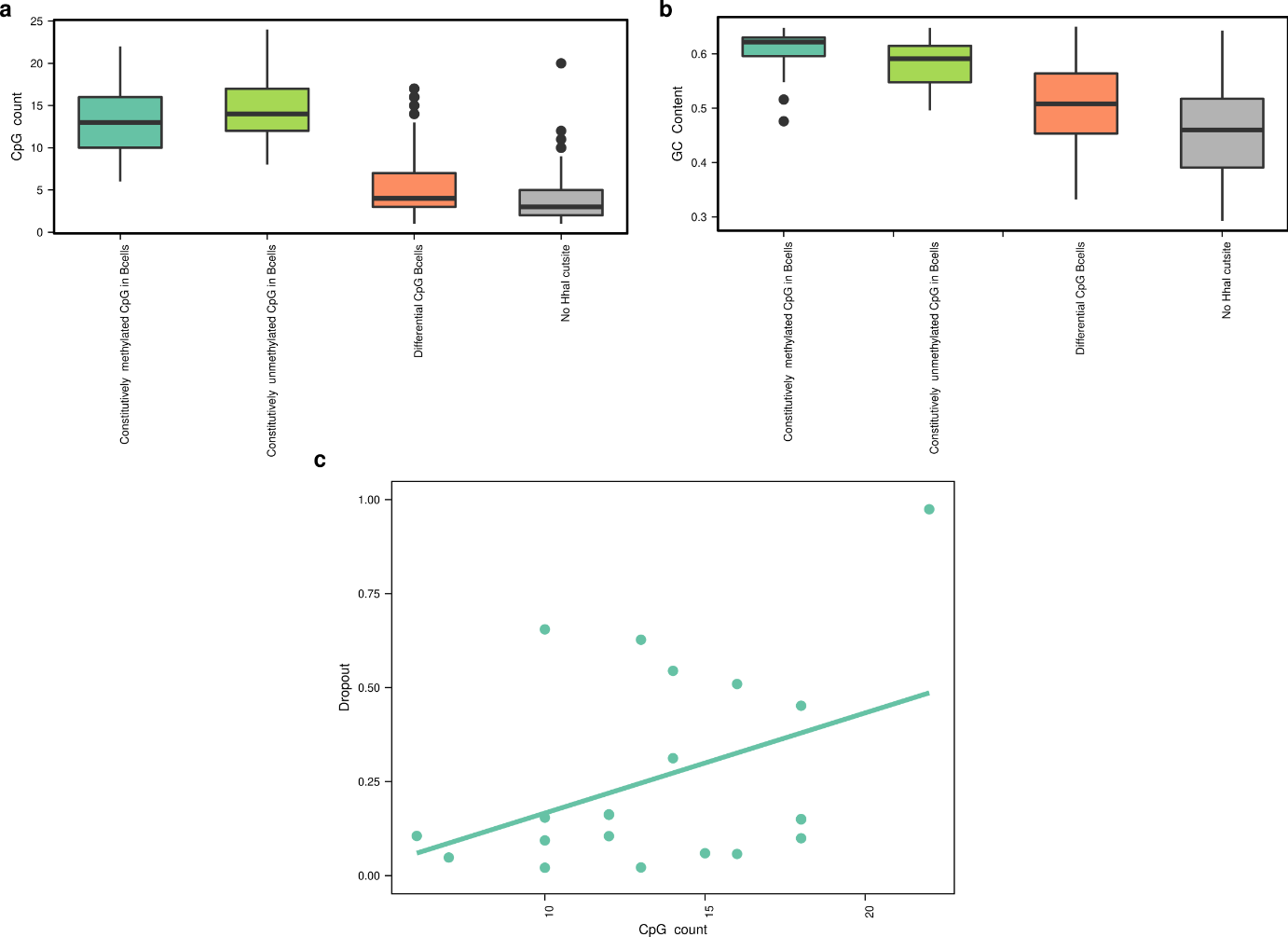


**Fig S5: Analysis of the effect of GC content on amplicon performance.** (**a**) CpG count and (**b**) GC content stratified according to the different amplicon classes. **c**. Relationship between CpG count and dropout rate in the bone marrow sample for the amplicons of class ‘constitutively methylated’. The solid line represents the least-squares linear regression line.


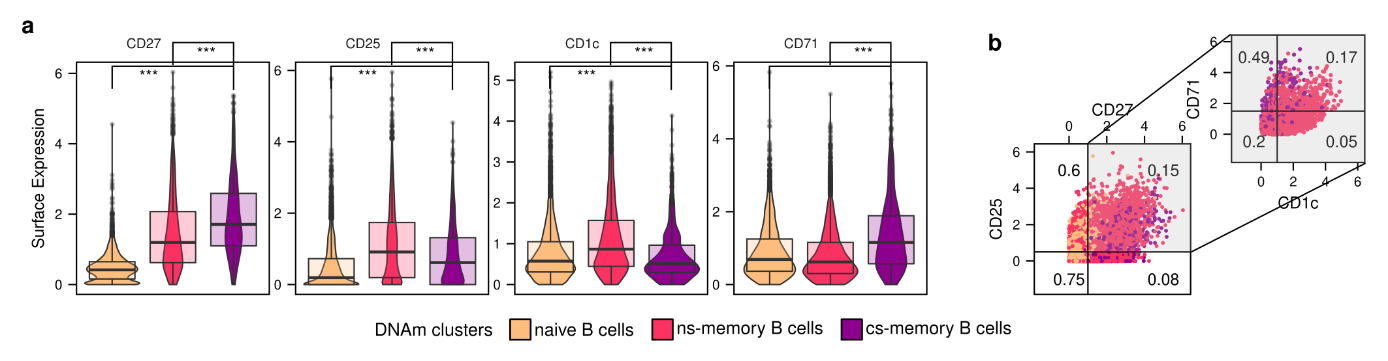


**Fig S6: Surface-protein expression of selected markers in the peripheral blood sample.** **a.** Boxplots with single-cell surface-protein expression levels. The markers were selected due to their expression profile in previous CITE-seq atlases (Triana *et al.*, 2021). From left to right: naive, non-switched (ns-) and class-switched (cs-) memory B cells. ***: Two-sided Wilcoxon test p-value<0.001. **b.** Scatterplot comparing surface-protein expression across different clusters. Numbers in quadrants represent the fraction of naive B cells (left panel) and cs-memory B cells (right panel) across all cells in the quadrant. Naive B cells (orange), non-switched memory B cells (ns-, red), class-switched memory B- cells (purple).


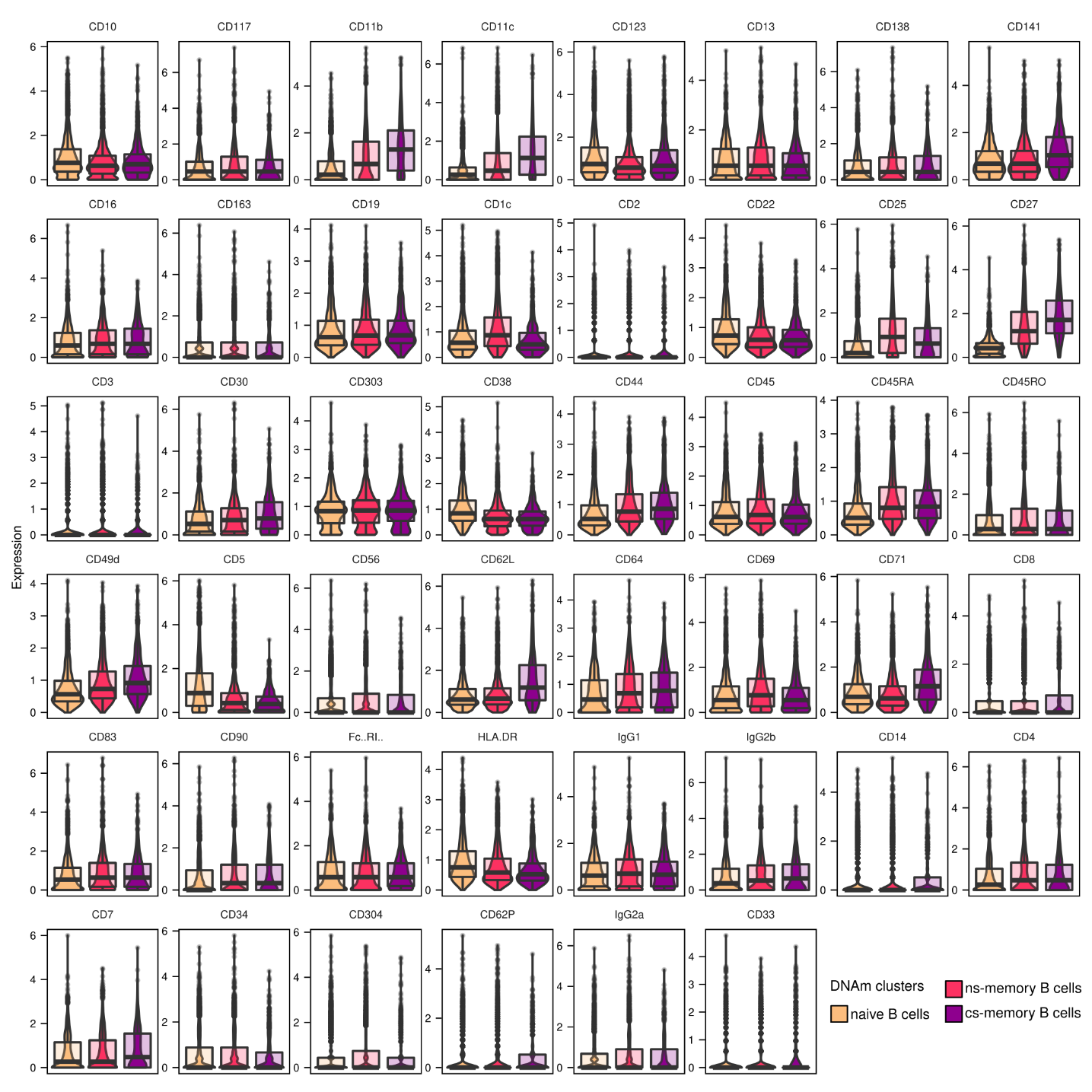


**Fig S7:** **Surface-protein expression in the peripheral blood sample.** Combined box- and violin-plot displaying the CLR-normalized expression for all 46 surface proteins analyzed using scTAM-seq across the three DNAm-based cell clusters. In each panel from left to right: naive, non-switched (ns-) and class-switched (cs-) memory B cells.


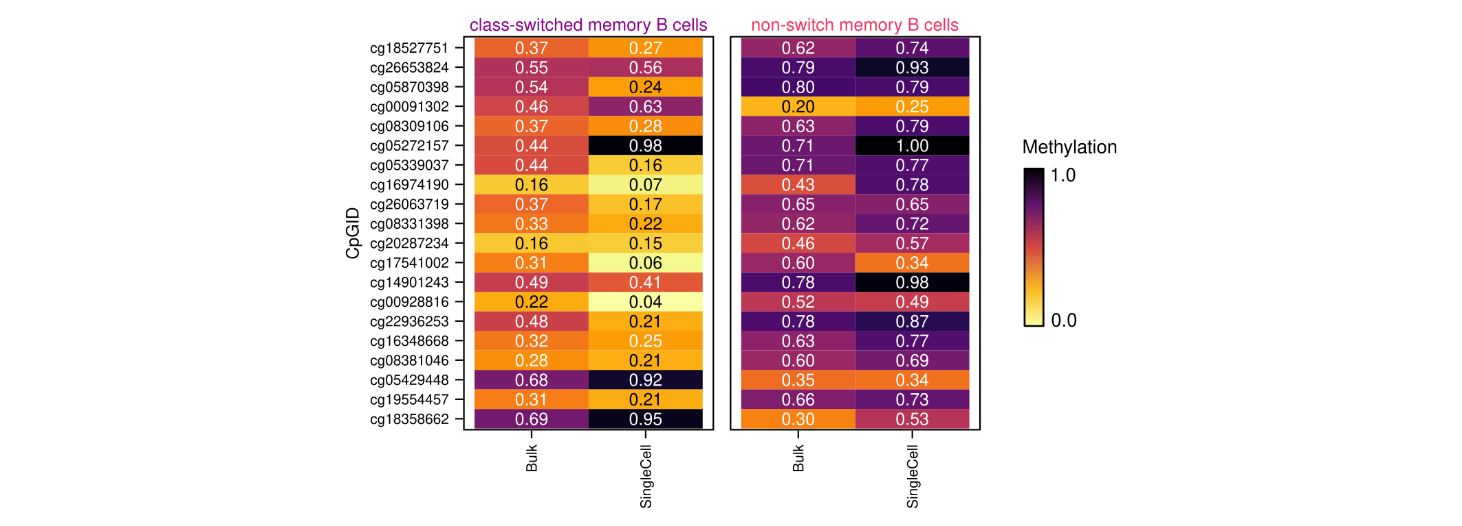
**Fig S8: Comparison of bulk and pseudo-bulk DNAm values for class-switched and non-switched memory B cells**. For the 20 CpGs with the highest mean difference in the bulk DNAm data, bulk and pseudo-bulk (=SingleCell) methylation values are listed.


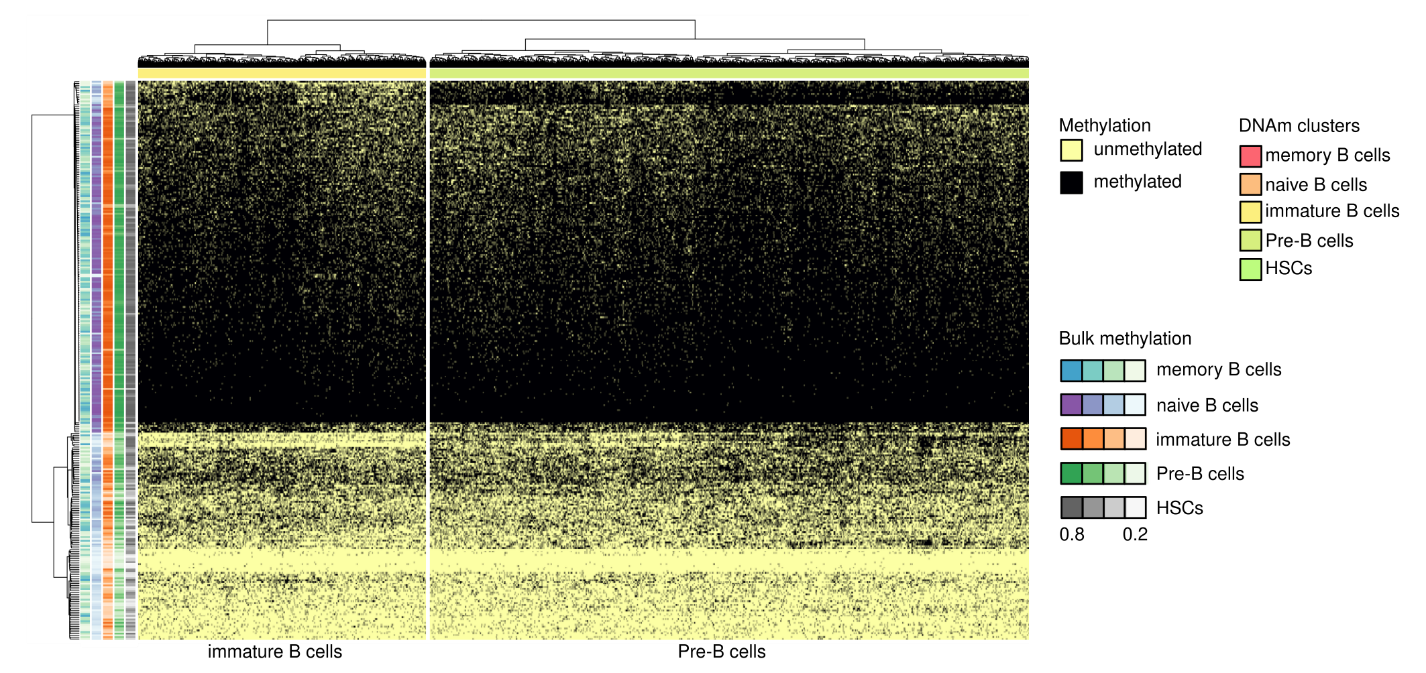


**Fig S9: Heatmap visualizing the DNAm states determined using scTAM-seq for the 2,366 cells in the pre/immature B-cell cluster.** The heatmap represents data across the 313 selected amplicons. Left part of the heatmap: immature B cell, right part: Pre-B cells.


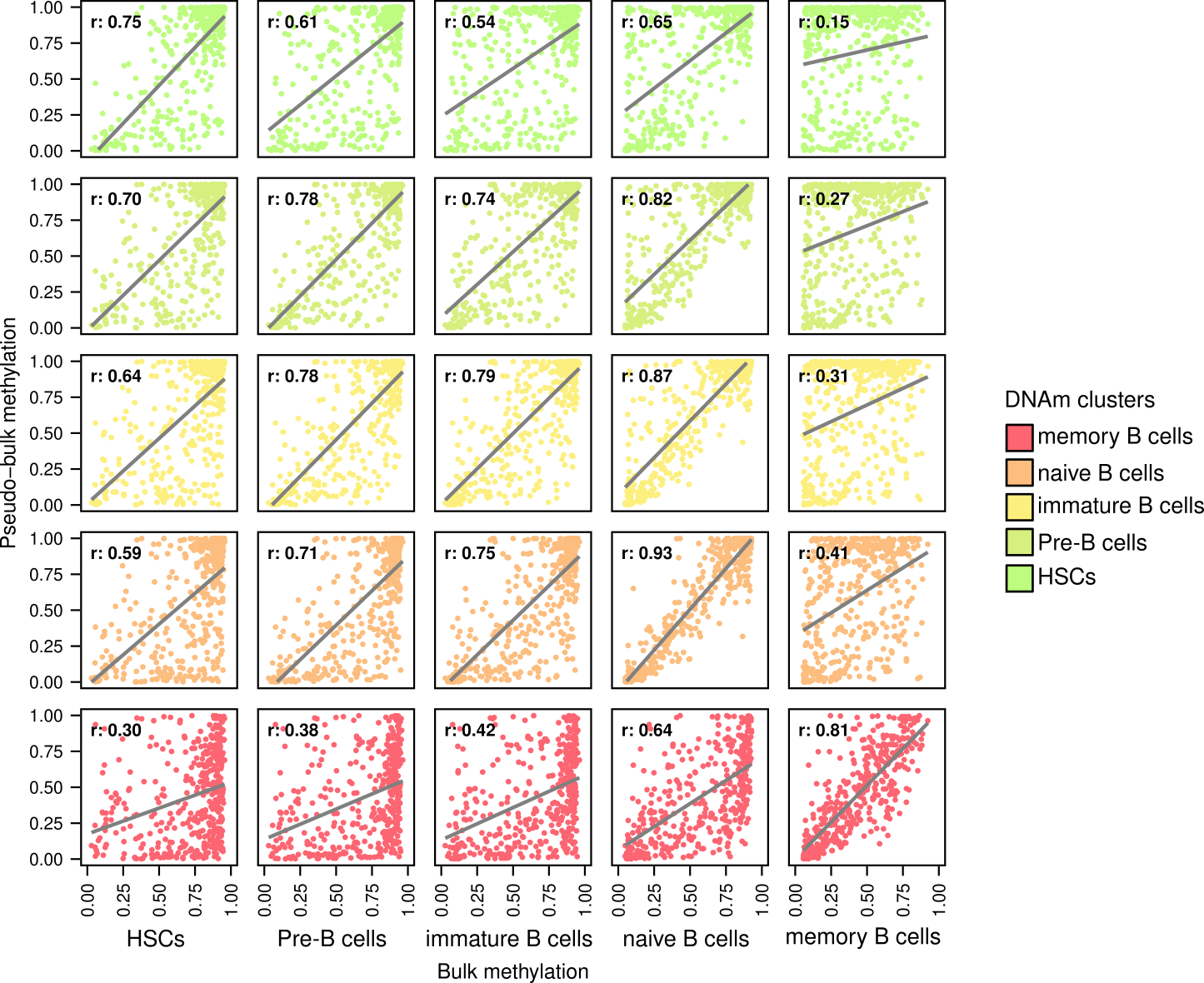


**Fig S10: Comparison of bulk (x-axis) and pseudobulk (y-axis) DNAm values for the five clusters defined on the bone marrow sample**. The comparisons include all 424 B-cell-differentiation amplicons. The r indicates Pearson’s correlation coefficient between x- and y-values and the solid line represents the least-squares linear regression line. The high correlation between the immature/pre-B cells and the naive B cell cluster can in part be explained by potential impurities in the respective bulk clusters (see Fig S11).


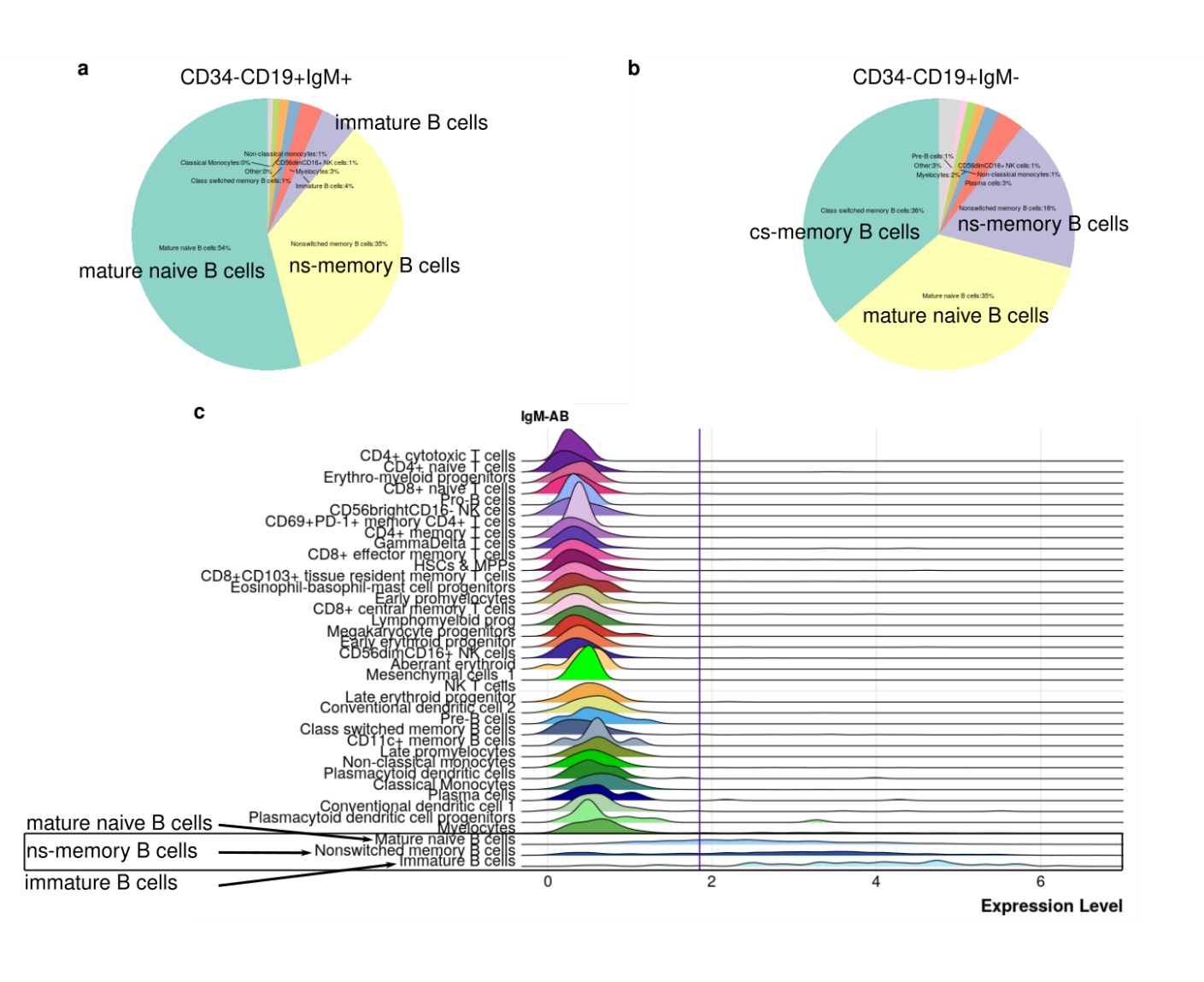

**Fig S11: Investigation of potential impurity induced in immature and pre-B-cells by the IgM-FACS-gate used in Kulis *et al*., 2015.** **a**: Distribution of cell types for CD34-negative, CD19-positive and IgM-positive cells in blood and bone marrow, computed from targeted CITE-seq with 197 antibodies (Triana *et al.*, 2021). **b**: Similar to a, for CD34-, CD19+ and IgM-negative cells. **c**: Expression of the IgM-surface antibody across all cell types in blood and bone marrow. The pictures have been obtained from the ABSeq-app (https://abseqapp.shiny.embl.de/) and are based on the data generated in Triana *et al*., 2021.


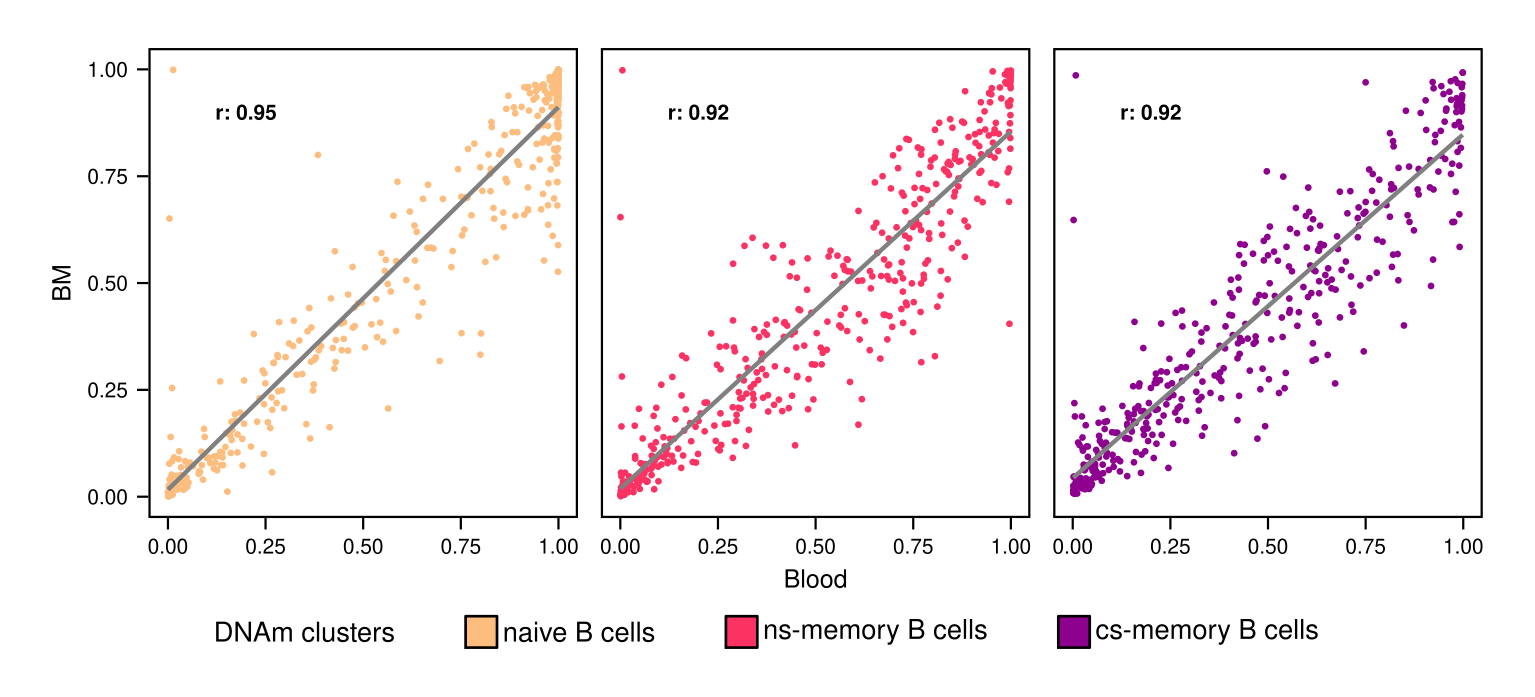
**Fig S12: Comparison between the pseudo-bulk DNAm values estimated in blood (x-axis) and in bone marrow (y-axis) for naive, cs- and ns-memory B cells.** Comparisons include all the 424 B-cell-differentiation amplicons. The r represents the Pearson correlation coefficient.


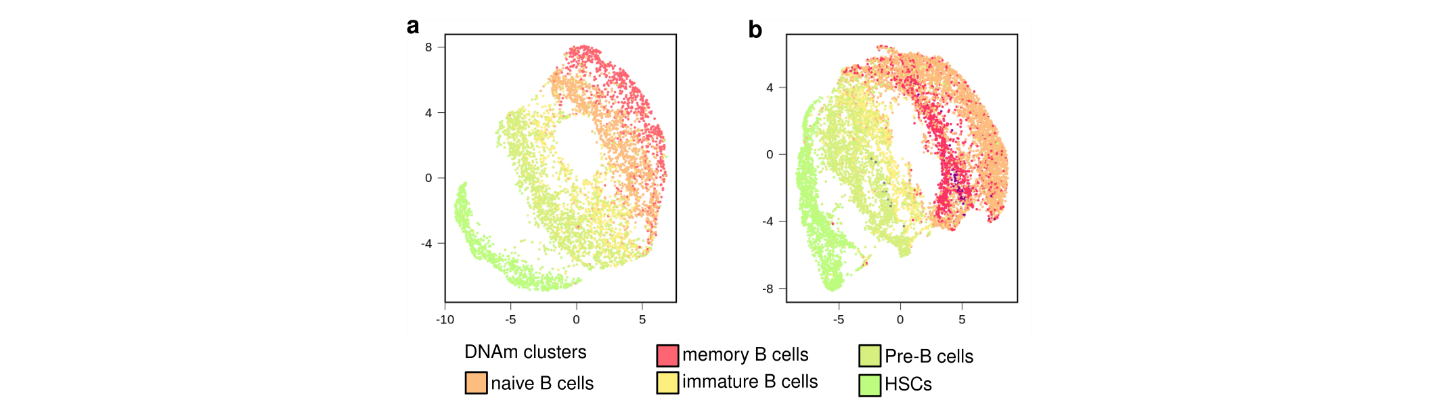


**Fig S13: Visualization of the surface-protein data for the bone marrow sample with the cells colored by the cell type labels inferred from the DNAm data.** Low-dimensional representation of the cell-surface protein expression as a UMAP (**a**). The surface protein expression data assayed using scTAM-seq was processed using Seurat (see Methods). **b**. Integration with cell-surface protein data from the CITE-seq reference atlas (Triana *et al*., 2021). Since scTAM-seq and the reference atlas share 33 surface markers, multi-modal nearest neighbors can be used to generate a joint low-dimensional representation of the two datasets (see Methods).


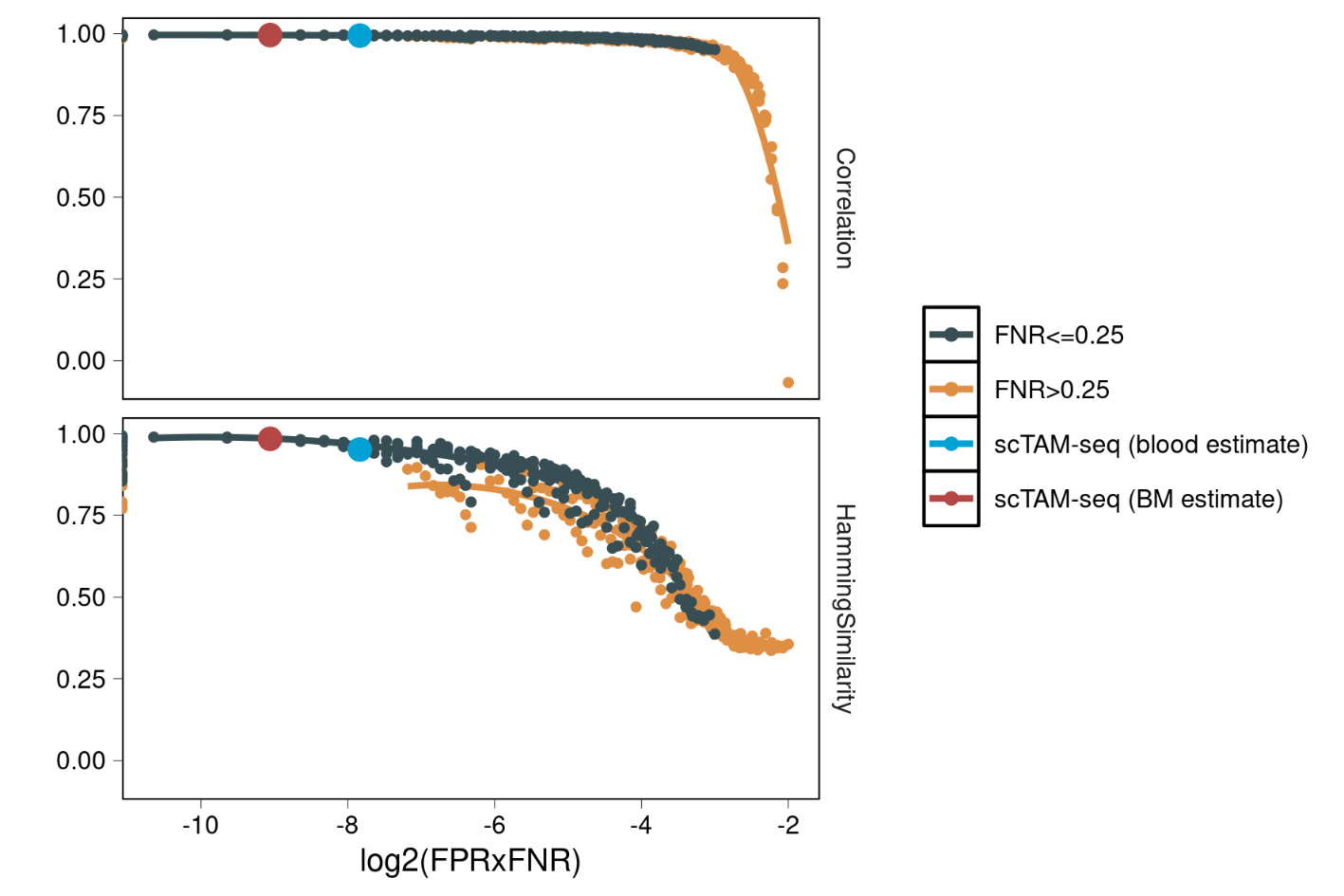

**Fig S14: Simulation of the effect of the false-positive and false-negative rate (FPR/FNR)**. Shown are the effect on the correlation between pseudo-bulk and bulk DNA methylation data (upper panel), and on the clustering accuracy measured using the Hamming similarity (i.e., the fraction of cluster assignments that agree, lower panel). The figure is stratified according to simulations with low and high FNR values (cutoff 0.25). scTAM-seq is marked in the plot with FNR=0.075 and FPR=0.025 (BM estimate, the lowest FPR value that we simulated in the grid of FPR/FNR) and with FNR=0.175/FPR=0.025 (blood estimate). The x-axis shows the logarithm with basis 2 for the product of the FPR and the FNR.


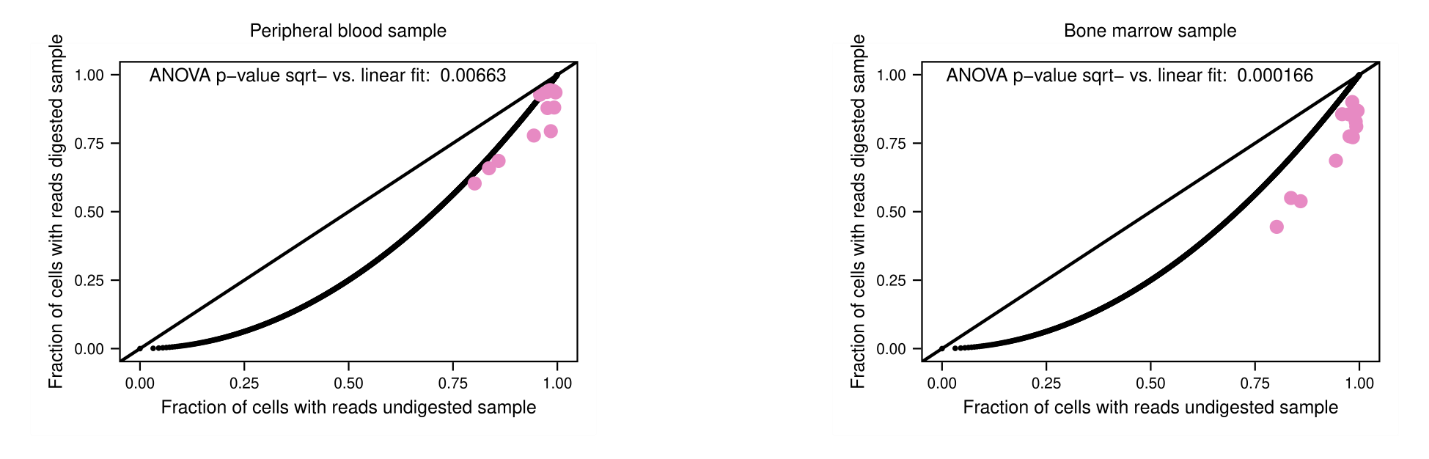
**Fig S15: Comparison of fraction of cells with reads for the 12 amplicons located within imprinting control regions.** These regions were obtained from Court *et al.,* 2014. The figure shows the fractions of cells with at least one sequencing read in the undigested (bone marrow, x-axis) versus the digested (peripheral blood/bone marrow, y-axis) sample. The solid, curved line indicates a square-root fit of the data, which assumes that only one of the alleles is methylated and captured after digestion, and the p-value results from an ANOVA comparing a linear versus the square-root fit.

**Fig S16: Bioanalyzer profiles of libraries**. Profiles from bone marrow digested (**a**, **b**) and undigested (**c**, **d**) samples are shown. **a**, **c**. scTAM-seq libraries, target peak: 460 bp. **b,** **d**. Surface-protein libraries, target peak: 260 bp.


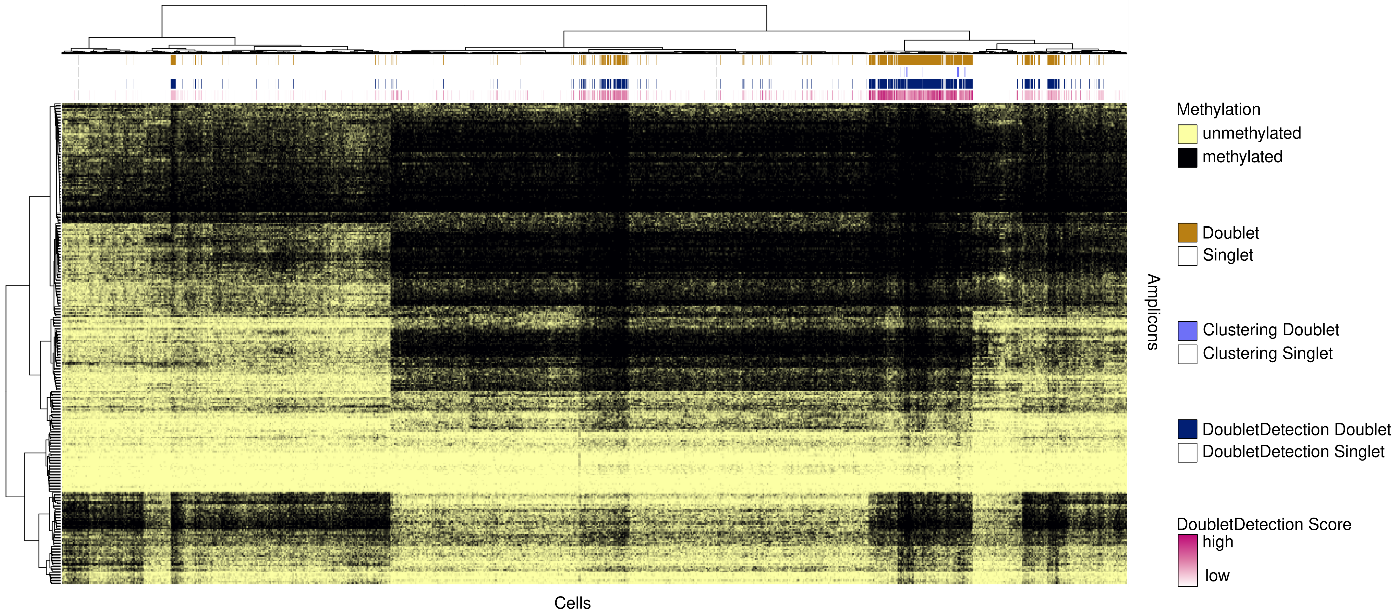


**Fig S17: Doublets as detected by the DoubletDetection software marked in the scTAM-seq heatmap for the digested blood sample.** Out of the 11,438 cells, 1,794 cells were marked as doublets by DoubletDetection. Additionally, we identified 61 potential doublets in a per-cluster analysis based on the number of features per cluster (Clustering doublets).
