# Supplementary material for: scTAM-seq enables targeted high-confidence analysis of DNA methylation in single cells
Source: Genome Biol. 2022 Oct 28;23:229. doi: 10.1186/s13059-022-02796-7 (PMC9615163; doi:10.1186/s13059-022-02796-7)
Supplement: Supplementary file 9 — Additional file 9. Review history. [file 13059_2022_2796_MOESM9_ESM.docx]

Review History

**First round of review**

**Reviewer 1**

**Were you able to assess all statistics in the manuscript, including the appropriateness of statistical tests used?**

Yes, and I have assessed the statistics in my report.

**Comments to author:**

The manuscript "scTAM-seq enables targeted high-confidence analysis of DNA methylation in single cells" by Bianchi et al., describes a novel high-throughput method for single-cell DNA methylation analysis. The presented method uses the Mission Bio Tapestri platform to perform PCR amplification of target regions containing a single CpG after digestion with HhaI, a methylation-sensitive restriction enzyme for which its digestion activity is completely blocked by DNA methylation. With this method, the authors investigated 424 CpGs that are known to be dynamically regulated during B cell differentiation. In addition, the authors investigated 140 control regions, consisting of 21 constitutively methylated CpGs, 32 constitutively unmethylated CpGs and 87 regions that do not contain HhaI recognition sequences. The authors applied scTAM-seq to the analysis of B cells from blood and bone marrow and combined this with the analysis of surface protein expression analysis. Using their method, they were able to resolve DNA methylation dynamics during B cell differentiation and revealed previously hidden intermediate differentiation states. Surface protein expression analysis further enabled the integration of single cell methylation data with available single cell transcriptome data generated with the CITE-seq method.

Overall, the present manuscript is well-written and thoroughly describes a novel method that will advance the field of single cell epigenomics and which might open the door towards clinically oriented applications e.g. in tumor profiling. I have only a few comments which I feel would be important to be addressed in order to fully assess the performance of this new method.

Major:

*	The data presented in this manuscript is based on two experiments, one analyzing B cells from blood and the other from bone marrow. In order to assess the reproducibility of the results, it would have been great to see how the results from a repeat experiment would look like and how the false-negative and false-positive rates behave across different experiments.

*	The authors report a median false-negative rate (FNR) of 7% and a false-positive-rate (FPR) of 0.2% at the level of single CpGs and single cells. I tried to figure out how exactly this was calculated but got a bit lost. I think, it would be good to have that explained clearly in the text. In addition, it would be great to show a simulation of how the FPR and FNR rates impact on the methylation calls both at the per CpG and at the per cell level, including the pseudo-bulks.

*	How did the authors deal with drop-outs for the binarized analysis? While from the methods section it is clear to me how the drop-out rate was used when calculating the pseudo-bulk methylation, it should be clarified if and how this was done for single cells. As stated above, it would be great to provide some data on how this affects the results. This is especially important as the authors used all CpGs with a FNR<0.25 as "high confidence" CpGs, meaning that quite a significant proportion of CpGs has could be wrongly assigned as unmethylated in almost 1 out of 4 cells….

*	As the methylation calls were binarized and a single read per amplicon was considered as methylated, I was asking myself how hemi-methylation (i.e. methylation of one out of two alleles) would impact on the binarized methylation calls. I would expect that hemi-methylation would result in a reduction of read counts at the respective locus. Is this visible from the raw-data and is there a way to identify hemi-methylated CpGs? Hemi-methylation (in contrast to heterogeneous methylation within a given cell population) might explain the overestimation of DNA methylation at some, but not all, loci presented in Suppl. Figure 5.

Minor:

*	Figure 1d: I suppose that this figure, similar to Figure 2a, shows binarized data clustered using binary distance and Ward's method, but this should be clearly indicated in the figure legend.

*	Figure 2c: a color map for the protein expression is missing.

*	The authors claim in the abstract that their method enables the analysis of DNA methylation, surface protein expression and somatic mutations. The latter seems to be a theoretical possibility for which the authors do not show any data in the present manuscript. This point should be clarified in the text.

*	Suppl. Figure 7: The pseudo-bulk methylation for both pre-B cells and immature B cells appeared to correlate best with the bulk methylation data derived from naïve B cells instead of their direct counterpart. Do the authors have any explanation for this observation (e.g. coverage of the bulk data; number of single cells from which the pseudo bulk has been generated, …)?

**Reviewer 2**

**Were you able to assess all statistics in the manuscript, including the appropriateness of statistical tests used?**

Yes, and I have assessed the statistics in my report.

**Comments to author:**

The authors describe scTAM-seq as a means of profiling the methylations tatus of up to 650 CG sites in up to 10k cells per experiment. The technique leverages methylation sensitive restriction enzymes to digest unmethylated amplicon targets in the MissionBio Tapestri platform. The technology appears to perform well, however some of the framing and language in the paper could be toned down. The authors present it as a means of achieving unprecedented resolution in DNA methylation dynamics; however, it is still a targeted approach, only assessing up to 650 sites - I would argue that it would not be capable of achieving the same resolution as something like Liu et al Nature 2021 (doi: 10.1038/s41586-020-03182-8) where 161 clusters were identified. The advantage of scTAM is that it is cheap - the Liu et al dataset was a multi-million USD experiment. The authors frame scTAM-seq as better with respect to discovery-based applications, which simply is not true; the real advantage (which is massively important) is that it is a cheap way to perform targeted DNA methylation analysis in high throughput - though comparing (and claiming it is favorable in information content to) genome-wide assays is not realistic. The technique stands well on its own and is an important advance without attempting to claim superiority to technologies that are not even doing the same thing.

The authors state that scWGBS methods only capture ~1-7% of CG sites; however, they do not cite snmC-seq2 which covers on the order of ~25% (Luo et. al. Nat. Comms. 2018). Also notably, even 1% of CG sites is still much larger than 650 at ~280,000. This does not discount the importance of targeting the most interesting CG sites, but the framing is attempting to compare targeted vs discovery-based efforts that are not all that similar. I suggest focusing on the power of scTAM's targeting ability and not trying to claim it can be used to replace unbiased discovery-based techniques.

While it is true that many CG sites are not all that informative; without unbiased genome-wide methods, the identification of CG sites that would be targeted by scTAM-seq is not possible. Bulk assays can be used to guide analysis, but it cannot be used to comprehensively identify variable CG sites across cell types within a population.

The authors should focus on the excessive cost of sequencing genome-wide single-cell methylation libraries - making it practically impossible for a typical research group to leverage the assay; whereas public atlas datasets of genome-wide single-cell methylation can be leveraged for groups to then identify targets for the cost-effective, low-sequencing-requirement scTAM-seq.

The method itself is very compelling and appears to perform quite well. It is a clever trick to capture meaningful DNA methylation information. However, unlike bisulfite methods there is only positive information (ie presence of methylation) whereas absence of methylation is indistinguishable from dropout. This is not a big issue since that is the case for most single-cell assays (e.g. RNA, ATAC, ADT/CITE, etc)

The authors provide a tool for CG site selection which is a nice addition.

Overall, analysis and methods are sound - it is an exciting technique that is readily available to any researcher with access to a Tapestri instrument. The only issue is framing - focusing on the real and impactful advances versus comparisons to genome-wide scWGBS techniques (that are not readily accessible an exceedingly expensive) would greatly benefit the paper.

**Authors Response**

**Point-by-point responses to the reviewers’ comments:**

**Reviewer 1:**

The manuscript "scTAM-seq enables targeted high-confidence analysis of DNA methylation in single cells" by Bianchi et al., describes a novel high-throughput method for single-cell DNA methylation analysis. The presented method uses the Mission Bio Tapestri platform to perform PCR amplification of target regions containing a single CpG after digestion with HhaI, a methylation-sensitive restriction enzyme for which its digestion activity is completely blocked by DNA methylation. With this method, the authors investigated 424 CpGs that are known to be dynamically regulated during B cell differentiation. In addition, the authors investigated 140 control regions, consisting of 21 constitutively methylated CpGs, 32 constitutively unmethylated CpGs and 87 regions that do not contain HhaI recognition sequences. The authors applied scTAM-seq to the analysis of B cells from blood and bone marrow and combined this with the analysis of surface protein expression analysis.

Using their method, they were able to resolve DNA methylation dynamics during B cell differentiation and revealed previously hidden intermediate differentiation states. Surface protein expression analysis further enabled the integration of single cell methylation data with available single cell transcriptome data generated with the CITE-seq method.

Overall, the present manuscript is well-written and thoroughly describes a novel method that will advance the field of single cell epigenomics and which might open the door towards clinically oriented applications e.g. in tumor profiling. I have only a few comments which I feel would be important to be addressed in order to fully assess the performance of this new method.

*We appreciate the nice summary and favorable overall assessment of our work by the reviewer and especially the fact that the reviewer agrees with us that scTAM-seq can be a substantial advance to the field of single-cell-epigenomics and will open the door for translational research. We thank the reviewer for raising points that can be improved, we addressed them point-by-point below.*

Major:

•	The data presented in this manuscript is based on two experiments, one analyzing B cells from blood and the other from bone marrow. In order to assess the reproducibility of the results, it would have been great to see how the results from a repeat experiment would look like and how the false-negative and false-positive rates behave across different experiments.

*The reviewer raises an important point and we would like to address the issues about reproducibility in details in the following. First, naive and memory B cells both exist in blood and in bone marrow extractions, with no major differences in gene expression (see e.g., Triana et al., Nature Immunology 2021). We can therefore treat these experiments as biological replicates and estimate a lower bound on the reproducibility of scTAM-seq. The correlation of pseudobulk DNA methylation values between bone marrow and blood is equal or higher than 0.92 for the three B-cell subpopulations (naive, non-switched, and class-switched memory B cells, see new Supplementary Figure 10, also shown below). These findings indicate a high level of concordance of the pseudo-bulk DNA methylation levels of biological replicates of three different B-cell subpopulations present in two different tissues, and using data generated in two independent scTAM-seq experiments.*

*“Supplementary Figure 10: Comparison between the pseudo-bulk DNA methylation values estimated on blood (x-axis) and on bone marrow (y-axis) for naive, cs- and ns-memory B cells for the 424 B-cell-differentiation amplicons. The r represents the Pearson correlation coefficient.”*

*Second, we have now further computed the FPR for the digested blood sample and the FNR for an undigested blood sample, to investigate the reproducibility of these values (new Supplementary Figure 2). The FPR was similar (0.001) to the bone marrow digested sample while the FNR was higher (0.163) than in the bone marrow undigested sample (0.067). The blood undigested sample had been processed first, before we ensured equal representations of the PCR duplicates performed to prepare the final libraries, resulting in a less balanced representation of amplicons (see Reviewer figure 1 below). In the final version of the protocol (used to prepare the undigested bone marrow sample and both digested samples), PCR duplicates were preformed and pooled at equal ratios to increase library complexity (Reviewer Figure 1). Furthermore, sequencing depth of the blood undigested sample was 28% lower, compared to the bone marrow undigested sample; together, these points can explain the higher FNR observed in the blood undigested sample. In the manuscript we now describe the range of FNRs observed. Further, in response to the reviewer’s second point below, we demonstrate that robust clustering and pseudobulk methylation estimates are obtained even with an FNR of 16% (Reviewer Figure 2). Notably, due to the above points, and the higher correlation observed for the control amplicons between the blood digested and bone marrow undigested control (see new Supplementary Figure 3), we exclusively used the bone marrow sample as control for selecting amplicons and computing pseudo-bulk values.*

*Reviewer Figure 1. Fraction of reads per amplicon in blood and bone marrow undigested samples. For each sample, amplicons are ranked by the fraction of total reads per amplicon.*

- The authors report a median false-negative rate (FNR) of 7% and a false-positive-rate (FPR) of 0.2% at the level of single CpGs and single cells. I tried to figure out how exactly this was calculated but got a bit lost. I think, it would be good to have that explained clearly in the text. In addition, it would be great to show a simulation of how the FPR and FNR rates impact on the methylation calls both at the per CpG and at the per cell level, including the pseudo-bulks.

*The reviewer raised a valid point. We rearranged the paragraph describing FPR and FNR in the main text and expanded on the explanation of how FPR and FNR were computed in the methods section of the manuscript as follows:*

*“As a first step, we computed false-positive rates (FPR) and false-negative rate (FNR) as follows; As the FPR represents the event when a read is present although the respective site is not methylated, we leveraged the 32 constitutively unmethylated amplicons in the digested sample to compute a FPR for each amplicon across all the cells. The FPR is defined as the fraction of cells that obtain at least one sequencing read across all cells for the constitutively unmethylated amplicons. Conversely, the FNR represents the probability of observing no sequencing read although there is either no digestion or the site is protected by methylation. We were particularly interested in the FNR of the 424 B-cell-differentiation amplicons and thus quantified their FNR in the undigested sample. Consequently, we computed the per-amplicon FNR as the fraction of cells not having a sequencing read across all cells.”*

*We thank the reviewer for suggesting simulation experiments to investigate the effect of FNR/FPR on the clustering accuracy and pseudo-bulk computation. To that end, we simulated 1,500 cells from three cell types (HSCs, naive, and memory B cells) for 100 CpGs. We used bulk DNA methylation values as Bernoulli success probabilities to simulate a binary single-cell DNA methylation matrix. Subsequently, we added noise by simulating false negatives and false positives, using increasing FPR and FNR in a range from 0 to 0.5. Finally, we compared the resulting clustering with the original clustering of cell types from the noise-free (FNR = FPR = 0), simulated single cell matrix (i.e., 500 cells of HSCs, naive, and memory B-cells each) and additionally compared the correlation between bulk and pseudo-bulk DNA methylation data generated using different combinations of FPR/FNR levels (Reviewer Figure 2). We observed that the FPR and FNR of scTAM-seq estimated from both the bone marrow and blood control indeed are low enough to reliably cluster cells and to estimate pseudo-bulk methylation values.*

*Reviewer Figure 2: Simulation of the effect of the false-positive and false-negative rate (FPR/FNR) on the correlation between pseudo-bulk and bulk DNA methylation data (upper panel), and on the clustering accuracy measured using the Hamming similarity (i.e., the fraction of cluster assignments that agree, lower panel). The figure is stratified according to simulations with low and high FNR values (cutoff 0.25). scTAM-seq is marked in the plot with FNR=0.075 and FPR=0.025 (BM estimate, the lowest FPR value that we simulated in the grid of FPR/FNR) and with FNR=0.175/FPR=0.025 (blood estimate). The x-axis shows the logarithm with basis 2 for the product of the FPR and the FNR.*

- How did the authors deal with drop-outs for the binarized analysis? While from the methods section it is clear to me how the drop-out rate was used when calculating the pseudo-bulk methylation, it should be clarified if and how this was done for single cells. As stated above, it would be great to provide some data on how this affects the results. This is especially important as the authors used all CpGs with a FNR<0.25 as "high confidence" CpGs, meaning that quite a significant proportion of CpGs has could be wrongly assigned as unmethylated in almost 1 out of 4 cells….

*We thank the reviewer for raising this point. Indeed, it is impossible to discern the lack of methylation from a dropout event at the level of single cells. As the reviewer points out, we can account for it while computing pseudo-bulk methylation values. With the simulation that we present in Reviewer Figure 1, we show that the observed false negative/false-positive DNA methylation rates do not strongly influence the analysis we conducted through clustering and pseudo-bulks. The dropout rate of scTAM-seq is considerably lower than a dropout rate that affects the overall clustering and pseudo-bulk values. Additionally, most of the 313 high confidence amplicons have dropout rates of less than 10% (n=238), while fewer have higher dropout rates (10-20%: 60, 20-25%: 15 amplicons). We like to point out that the FNR in scRNA-seq, scATAC-seq and other scMET methods is considerably higher than 25% (see for example Svensson et al., Nature Methods 2017 for computation of the molecular detection limit in scRNA-seq). Finally, we would like to comment that the definition of “high-confidence CpGs” is not fixed, and it can be adapted according to the requirements of the user for downstream applications. For instance, to perform single-CpG analysis one can choose to use only amplicons with an FNR<0.10 or FNR <0.05, substantially decreasing the chance of wrongly assigning a CpG as unmethylated at the single-cell level.*

•	As the methylation calls were binarized and a single read per amplicon was considered as methylated, I was asking myself how hemi-methylation (i.e. methylation of one out of two alleles) would impact on the binarized methylation calls. I would expect that hemi-methylation would result in a reduction of read counts at the respective locus. Is this visible from the raw-data and is there a way to identify hemi-methylated CpGs? Hemi-methylation (in contrast to heterogeneous methylation within a given cell population) might explain the overestimation of DNA methylation at some, but not all, loci presented in Suppl. Figure 5.

*The reviewer raises an important and valid point. Since we were also intrigued by this question when designing the panel, we included 12 imprinted regions identified in Court et al. (2014) into our panel. In these regions, after digestion, only one allele is available for amplification, whereas two alleles are available in the non-digested sample. Accordingly, the number of cells receiving a sequencing read in the digested sample grows with the square of the number of cells receiving a sequencing read in the undigested sample (new Supplementary Figure 12 and below). By contrast, for fully methylated amplicons, dropout is linearly correlated in the digested and non-digested experiment.*

*“Supplementary Figure 12: Comparison of fraction of cells with reads for the 12 amplicons located within imprinting control regions obtained from Court et al., 2014 for the peripheral blood and bone marrow sample. The figure shows the fractions of cells with at least one sequencing read in the undigested (bone marrow, x-axis) versus the digested (peripheral blood/bone marrow, y-axis) sample. The solid, curved line indicates a square-root fit of the data, which assumes that only one of the alleles is methylated and captured after digestion, and the p-value results from an ANOVA comparing a linear versus the square-root fit.”*

*While these results indicate that scTAM-seq is affected by hemi-methylation, we were not able to “identify” the imprinted regions in an unsupervised manner, hampered by the fact that 12 amplicons are insufficient to develop and validate such models. In the future, we are considering to design panels specifically targeted towards imprinted regions to investigate this question in more detail.*

*We agree with the reviewer that allele- and strand-specific methylation might have an effect on the pseudo-bulk computation. In fact, if the amplicon performs well, we would expect allele/strand-specific methylation to be visible as a fully methylated state in scTAM-seq pseudobulk data and an intermediate methylation in bulk. We have mentioned the possibility of detecting hemi-methylated states in the Discussion section of the revised manuscript:*

*“Since scTAM-seq exhibits a low FNR and FPR, we envision that it can also be used to further investigate imprinted regions, as well as other regions harbouring allele- and strand-specific methylation (Suppl. Fig. 12). Ultimately, single-cell DNA methylation values can help to discern cellular heterogeneity from allele-specific methylation, which in bulk data can only be achieved in special situations where SNPs are located on the same sequencing read. Conversely, allele- and strand-specific methylation might lead to an overestimation of pseudobulk methylation values by scTAM-seq.”*

Minor:

•	Figure 1d: I suppose that this figure, similar to Figure 2a, shows binarized data clustered using binary distance and Ward's method, but this should be clearly indicated in the figure legend.

*We appreciate the comment by the reviewer and added the corresponding note to the figure legend. Indeed, the clustering was also computed using the binary distance and Ward’s method.*

•	Figure 2c: a color map for the protein expression is missing.

*We thank the reviewer for spotting the missing annotation and added the corresponding legend to Figure 2c.*

•	The authors claim in the abstract that their method enables the analysis of DNA methylation, surface protein expression and somatic mutations. The latter seems to be a theoretical possibility for which the authors do not show any data in the present manuscript. This point should be clarified in the text.

*We have now removed this statement from the abstract and instead discuss it in the discussion section of the manuscript:*

*“Of note, the Mission Bio Tapestri platform was originally developed for profiling somatic mutations in cancer at single cell resolution [17]. These abilities can readily be integrated into scTAM-seq. Sites of potential somatic mutations can regularly be included into the amplicon panel and thus profiled together with the cell-type-specific DNA methylation state, as long as there is no HhaI cut site in the amplicon. Thereby, it has the potential to uncover clonal and sub-clonal development of tumors from the combined angle of somatic mutation, DNA methylation and protein surface proteins. It thus allows us to characterize the surface protein phenotypes of cells defined by specific DNA methylation patterns, and to develop FACS schemes for newly identified cell types [20].”*

•	Suppl. Figure 7: The pseudo-bulk methylation for both pre-B cells and immature B cells appeared to correlate best with the bulk methylation data derived from naïve B cells instead of their direct counterpart. Do the authors have any explanation for this observation (e.g. coverage of the bulk data; number of single cells from which the pseudo bulk has been generated, …)?

*We would like to thank the reviewer for noticing this issue. While investigating the high correlation between immature/pre-B-cells and naive B cells, we realized that in the original publication from which we obtained the bulk methylation data (Kulis et al., 2015), immature/pre-B-cells were differentiated based on IgM surface expression. However, IgM is also substantially expressed on naive and memory B cells that can be found in bone marrow extractions (see Reviewer Figure 3, https://abseqapp.shiny.embl.de/, Triana et al., 2021). Thus, the bulk methylation data for immature/pre-B-cells could potentially be contaminated with naive B cells explaining part of the observed correlation. Further discrepancies might be explained by the fact that the bulk data was obtained from fetal bone marrow, which may display differences in comparison to adult bone marrow that was analyzed by scTAM-seq.*

*Reviewer Figure 3: Investigation of potential impurity induced in immature and pre-B-cells by the IgM-FACS-gate used in Kulis et al., 2015. a: Distribution of cell types for CD34-negative, CD19-positive and IgM-positive cells in blood and bone marrow, computed from targeted CITE-seq with 197 antibodies. b: Similar to a, for CD34-, CD19+ and IgM-negative cells. c: Expression of the IgM-surface antibody across all cell types in blood and bone marrow. The pictures have been obtained from the ABSeq-app (https://abseqapp.shiny.embl.de/) and are based on the data generated in Triana et al., 2021.*

**Reviewer 2:**

- The authors describe scTAM-seq as a means of profiling the methylations tatus of up to 650 CG sites in up to 10k cells per experiment. The technique leverages methylation sensitive restriction enzymes to digest unmethylated amplicon targets in the MissionBio Tapestri platform. The technology appears to perform well, however some of the framing and language in the paper could be toned down. The authors present it as a means of achieving unprecedented resolution in DNA methylation dynamics; however, it is still a targeted approach, only assessing up to 650 sites - I would argue that it would not be capable of achieving the same resolution as something like Liu et al Nature 2021 (doi: 10.1038/s41586-020-03182-8) where 161 clusters were identified. The advantage of scTAM is that it is cheap - the Liu et al dataset was a multi-million USD experiment. The authors frame scTAM-seq as better with respect to discovery-based applications, which simply is not true; the real advantage (which is massively important) is that it is a cheap way to perform targeted DNA methylation analysis in high throughput - though comparing (and claiming it is favorable in information content to) genome-wide assays is not realistic. The technique stands well on its own and is an important advance without attempting to claim superiority to technologies that are not even doing the same thing.

*We thank the reviewer for providing a useful perspective on the strengths of our method and acknowledge that the comparison with discovery-based, genome-wide methods was not sufficiently balanced. We fully agree with the reviewer that scTAM-seq has important applications on its own and can serve as an important complement to genome-wide methods. Furthermore, while we agree that genome-wide methods have advantages in a discovery setting over scTAM-seq, provided that a sufficient number of cells can be profiled, we politely disagree that scTAM-seq cannot be used for discovery. Variably methylated CpGs can be identified a priori from bulk data and leveraged at single-cell level to identify complex cellular substructures, thus enabling discovery of subpopulations masked in bulk data using scTAM-seq, as we demonstrated in the setting of B-cell differentiation. We now provide a more balanced discussion of these points, both in the introduction and in the discussion section of the manuscript:*

*Introduction:*

*“Bulk DNAm data is available for most human tissues and cell types [10], and can be used to identify CpGs with variable methylation [11]. Additionally, single-cell, genome wide reference methylation maps are emerging, and serve as a rich resource for identifying variably methylated CpGs [12]. However, generating these maps are multimillion-dollar efforts that need to be complemented by cheaper methods accessible to individual investigators and for future clinical applications. Targeted approaches that focus sequencing on variable CpGs can efficiently dissect intra-tissue DNAm heterogeneity and complement single-cell genome-wide methods. In most human tissues and cell types, targeted methods can capture a large fraction of the variation observed in whole genome data (Suppl. Fig. 1), but have so far been limited to a throughput of less than 100 cells per experiment, and less than 60 investigated CpGs per cell [13–15]. To fill the existing gap of high-throughput, targeted single-cell DNA methylation methods, we developed scTAM-seq, a targeted bisulfite-free method for profiling up to 650 CpGs in up to 10,000 cells.”*

*Discussion:*

*“We present scTAM-seq as a targeted, cost-effective, scDNAm method with an FPR of less than 0.2% and an FNR as low as 7%, depending on the amplicon GC content and sequencing depth. As scTAM-seq is a targeted method, only up to 650 CpGs of the 28 million CpGs in the human genome can be analyzed. This might lead to a decreased ability for de novo cell type discovery in comparison to large-scale projects employing genome-wide scDNAm methods [29]. On the other hand, genome-wide approaches for scDNAm profiling largely suffer from limited cellular throughput and high data sparsity. Targeted methods serve as complementary approaches to genome-wide (single-cell) DNAm profiling methods, since the reduction in data sparsity allows for a more detailed analysis of selected CpGs in a large number of cells, rather than having to bin single-cell methylation states across genomic windows. Additionally, for most tissues and cell types, bulk DNAm data is readily available, revealing that only few CpGs are variable across cell differentiation (Suppl. Fig. 1). Thus, targeting only a few, highly variable CpGs is promising to reveal DNAm heterogeneity across cellular differentiation at unprecedented accuracy. Of note, the Mission Bio Tapestri platform has recently been extended to cover 1,000 amplicons, which increases the coverage of scTAM-seq even further. The selection of potentially interesting CpGs for scTAM-seq is facilitated by tools for the identification of variably methylated sites from bulk data [11]. A pipeline automating the selection of such target sites for scTAM-seq is available at https://github.com/veltenlab/CpGSelectionPipeline.”*

- The authors state that scWGBS methods only capture ~1-7% of CG sites; however, they do not cite snmC-seq2 which covers on the order of ~25% (Luo et. al. Nat. Comms. 2018). Also notably, even 1% of CG sites is still much larger than 650 at ~280,000. This does not discount the importance of targeting the most interesting CG sites, but the framing is attempting to compare targeted vs discovery-based efforts that are not all that similar. I suggest focusing on the power of scTAM's targeting ability and not trying to claim it can be used to replace unbiased discovery-based techniques.

*We modified the discussion of targeted vs. genome-wide methods, as detailed in the response to the previous point. We apologize for not including the reference to snmC-seq2, which is arguably among the highest-performance scDNAm methods available. We have included it in the revised version*

*“Available techniques with a cellular throughput of more than 100 cells produce very sparse datasets, where only 1-7% of the investigated CpGs are randomly covered in a single cell [1–6]. Notably, recent progress in scDNAm methods substantially increased the number of CpGs to up to 30% [7], but still requires prohibitive sequencing effort to substantially reduce the dropout rate.”*

- While it is true that many CG sites are not all that informative; without unbiased genome-wide methods, the identification of CG sites that would be targeted by scTAM-seq is not possible. Bulk assays can be used to guide analysis, but it cannot be used to comprehensively identify variable CG sites across cell types within a population.

*Sequencing reads in bulk bisulfite sequencing assays comprise single-molecule information. Quantifying the heterogeneity in sequencing patterns from this data can lead to the identification of variable DNA methylation patterns that can represent cell types and states (https://doi.org/10.1093/nar/gkaa120). However, we agree with the reviewer that if these cellular states are rare, heterogeneous patterns in bulk data will not be sufficient to identify them, and that bulk assays are not as comprehensive as single-cell, genome-wide methods for their identification. Nevertheless, we envision that scTAM-seq can be used to further characterize the cellular state substructures associated with variably methylated sites in bulk data. Targeting single-cell DNA methylation analyses to these heterogeneous CpG sites could enhance the discovery of cell types that otherwise may remain hidden.*

- The authors should focus on the excessive cost of sequencing genome-wide single-cell methylation libraries - making it practically impossible for a typical research group to leverage the assay; whereas public atlas datasets of genome-wide single-cell methylation can be leveraged for groups to then identify targets for the cost-effective, low-sequencing-requirement scTAM-seq.

*We agree with the reviewer on that point and have included it in introduction and discussion (see also the reviewer’s first point).*

- The method itself is very compelling and appears to perform quite well. It is a clever trick to capture meaningful DNA methylation information. However, unlike bisulfite methods there is only positive information (ie presence of methylation) whereas absence of methylation is indistinguishable from dropout. This is not a big issue since that is the case for most single-cell assays (e.g. RNA, ATAC, ADT/CITE, etc)

*We agree with the reviewer that scTAM-seq only provides a positive readout on a per-experiment basis, but due to the sophisticated control samples and amplicons that we incorporated into our study, we feel that we can confidently discern technical dropouts from lack of DNA methylation. To further stress this point, we now simulated DNA methylation data with different dropout levels in Reviewer Figure 2 and found that the FNR/FPR of scTAM-seq is below those that would affect pseudo-bulk methylation values and overall cell type clustering. We would also refer to the answer to Reviewer 1’s second point on the effect of FNR methylation calls on the clustering.*

- The authors provide a tool for CG site selection which is a nice addition.

*We appreciate that the reviewer points out the importance of the CpG selection for scTAM-seq. We will continue to develop the code to select those CpGs.*

- Overall, analysis and methods are sound - it is an exciting technique that is readily available to any researcher with access to a Tapestri instrument. The only issue is framing - focusing on the real and impactful advances versus comparisons to genome-wide scWGBS techniques (that are not readily accessible an exceedingly expensive) would greatly benefit the paper.

*We thank the reviewer for the favorable overall assessment of our work and agree that some of the formulations in the original paper were not well balanced. To that end, we re-formulated parts of the manuscript as stated above.*

**Second round of review**

**Reviewer 1**

Thanks a lot for nicely addressing my questions regarding the scTAM-seq manuscript and congratulations to this great manuscript! I am fully satisfied with the answers and the revised version of the manuscript. The only thing I would like to suggest in addition, is to consider incorporating Reviewer Figure 2 (showing the results of the FNR & FPR simulations) in the Supplements of the manuscript as this might provide useful information for the readership.
